# Bi-layer PVA/sodium alginate active films reinforced with clays and functionalized with *Nigella sativa* essential oil: enhanced stability, controlled release, molecular interaction insights, and fresh beef shelf-life extension

**DOI:** 10.3389/fbioe.2026.1937408

**Published:** 2026-09-28

**Authors:** Abderrahim Bouftou, Adil El meskine, Amine Joual, Ayoub Amahrous, Othman Tigri, Redouane Messaoudi, Anas Salihi, Fatima Lakhdar, Mustapha Lamine, Elmouloudi Sabbar, Rabiaa Fdil, Sanaa Majid

**Affiliations:** 1 Laboratory of Materials, Process, and Environmental Engineering, Faculty of Sciences Aïn Chock, Hassan II University of Casablanca, Casablanca, Morocco; 2 Laboratory of Materiel’s Physical Chemicals, Department of Chemistry, Faculty of Sciences, University Chouaib Doukkali, El Jadida, Morocco; 3 Laboratory of Applied Chemistry and Environment, Hassan 1st University Faculty of Science and Technology, Settat, Morocco; 4 Laboratory of Nuclear, Atomic, Molecular, Mechanical, and Energy Physics, Faculty of Sciences, Chouaib Doukkali University, El Jadida, Morocco; 5 Bioorganic Chemistry Team, Faculty of Sciences, Chouaïb Doukkali University, El Jadida, Morocco; 6 Laboratory of Marine Biotechnology and Environment, Faculty of Sciences, Chouaib Doukkali University, El Jadida, Morocco; 7 Laboratory of Mechanics and High Energy Physics, Faculty of Sciences Aïn Chock, Hassan II University of Casablanca, Casablanca, Morocco

**Keywords:** active coating, beef, food safety, nanoclay, Nigella sativa essential oil

## Abstract

In this study, active bilayer films based on PVA-SA, activated with *Nigella sativa* essential oil (NSEO) and stabilized with nanoclays (bentonite and LDH), were successfully developed and characterized. The incorporation of the essential oil-clay systems significantly improved the physicochemical properties of the films. The developed films exhibited satisfactory hydrophobicity, with a water contact angle of 53.8°, while water solubility and water absorption capacity were significantly reduced. Mechanical properties were also improved due to the plasticizing effect of the essential oil, as confirmed by DSC analysis, which showed a decrease in the glass transition temperature to 48.93 °C. Improved thermal stability was also evidenced by TGA analysis. The developed films exhibited strong antioxidant activity, with a DPPH radical scavenging activity of 59%, as well as significant antibacterial activity against *Escherichia coli* and *Staphylococcus aureus*, with inhibition zone diameters of 21 mm and 20.5 mm, respectively. Furthermore, the barrier properties were enhanced by improved UV-shielding ability and a reduced water vapor transmission rate of 45 g m^-2^·h^-1^ compared with the PVA-SA-EO film. The films also enabled a controlled release of essential oil bioactive compounds was observed up to 14 days. Molecular docking, rheological, FTIR, and XRD analyses suggested the existence of interactions between the polymer matrix, nanoclays, and the major constituents of the essential oil. The developed films were subsequently applied as spray coatings for the preservation of refrigerated beef. The coatings effectively preserved beef quality by limiting the increases in pH, lipid oxidation (TBARS), protein degradation (TVB-N), color deterioration, and weight loss during 10 days of storage at 4 °C. Overall, the developed PVA-SA-EO-clay coatings represent a promising, sustainable, and environmentally friendly strategy for extending shelf life, reducing food waste, and improving the safety of protein-rich foods.

## Graphical Abstract

**Figure:**
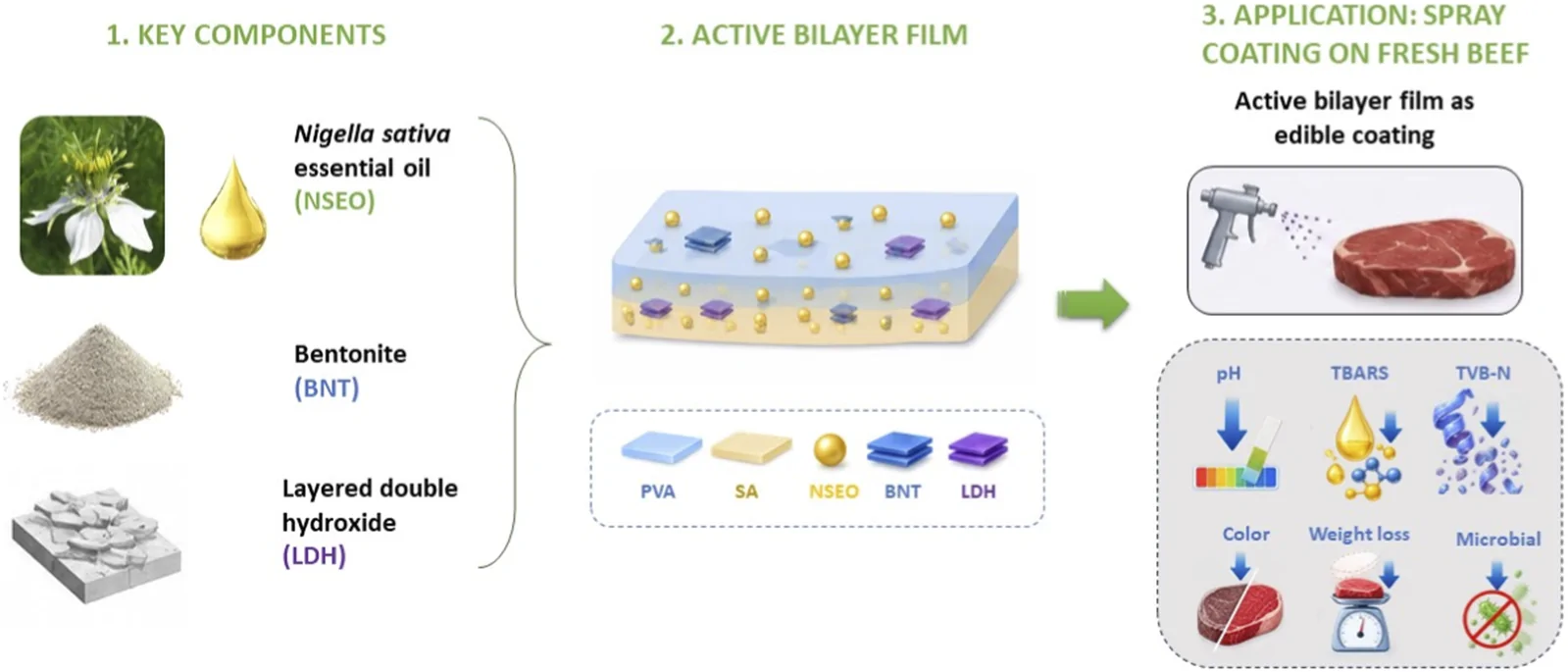

## 1 Introduction

Spoiled meat is considered one of the major sources of foodborne illnesses due to its high susceptibility to deterioration and its relatively short shelf life. Among the main factors responsible for meat spoilage is microbial proliferation, which leads to rapid deterioration of the microbiological, physicochemical, and sensory quality of meat products (Han et al., 2014). Therefore, numerous strategies have been developed to inhibit microbial growth on meat surfaces, including the immersion or spraying of antimicrobial compounds directly onto the product. These approaches aim to delay spoilage processes and extend the shelf life of meat products (Yu H. et al., 2026).

In recent years, growing consumer concerns regarding human health and environmental sustainability have driven the search for natural, safe, and biodegradable alternatives for food preservation. In this context, plant extracts and essential oils have attracted considerable attention because of their remarkable antimicrobial and antioxidant properties. Numerous studies have demonstrated their effectiveness in inhibiting microbial growth and delaying oxidative reactions responsible for food deterioration (Doha et al., 2025; Anthomelides et al., 2026; Purbey et al., 2026).

However, despite their significant bioactive potential, essential oils exhibit several limitations that restrict their direct application in food systems. Their high volatility, low stability, and sensitivity to environmental conditions often require the use of relatively high concentrations to achieve satisfactory preservation effects. Furthermore, the direct incorporation of essential oils into foods may impart intense aromas and flavors that can negatively affect consumer acceptability (Xu et al., 2026). To overcome these limitations, considerable research efforts have focused on the incorporation and stabilization of essential oils within biodegradable polymeric matrices designed for active food packaging and coating applications. Nevertheless, conventional polymeric films are often unable to provide long-term protection for these volatile compounds. Consequently, several advanced encapsulation technologies have been developed, including emulsification (Rajaei et al., 2026), emulsification-adsorption (Bouftou et al., 2024b), coacervation (Koç et al., 2026), ionic gelation (Rajabi et al., 2026), inclusion complexation (Yu Y. et al., 2026), spray drying (Ashaq et al., 2025), electrospinning (Mathew et al., 2026), and other related techniques. These strategies improve the stability of essential oils, protect their bioactive compounds against premature degradation, and enable their controlled release during storage. As a result, enhanced antimicrobial and antioxidant performance can be achieved even at low concentrations, promoting the development of innovative and sustainable food preservation systems (Hedayati et al., 2023).

Despite their effectiveness, these encapsulation approaches may involve relatively high costs and the use of specialized or expensive chemical agents, which can limit their scalability and practical application. Therefore, the present study explores the use of clay-based nanomaterials, namely, bentonite (BNT) and layered double hydroxide (LDH), as potentially cost-effective alternatives for stabilizing essential oil within the polymeric matrix. These materials were selected not only because of their reported adsorption capacity and favorable interactions with various compounds, but also because of their reported low toxicity and their established use in several fields beyond food packaging (Lu et al., 2025; Zheng et al., 2026). Moreover, the selection of BNT and LDH enables a comparative investigation of two structurally distinct clay materials, one naturally occurring (BNT) and the other synthetically prepared (LDH), and their respective effects on essential oil stabilization, film properties, and controlled release behavior.

The extensive use of non-biodegradable plastic food packaging films has significantly contributed to environmental pollution problems. Therefore, considerable research efforts have been directed toward the development of biodegradable and environmentally friendly food packaging materials, particularly those based on polyvinyl alcohol (PVA) and sodium alginate (SA). These biodegradable polymers exhibit excellent film-forming properties, along with good solubility, low oxygen permeability, high chemical stability, transparency, and strong adhesion characteristics. However, their individual use in food packaging applications remains limited due to several drawbacks, including their low antioxidant activity, lack of intrinsic antibacterial properties, high water sensitivity resulting from their hydrophilic nature, and relatively poor mechanical performance (Rusliman et al., 2026; Shirdel and Zeinali, 2026). To overcome these limitations, PVA and sodium alginate have been combined in food packaging applications to take advantage of their complementary properties and improve the overall performance of the resulting films. In the present study, a bilayer architecture was selected to spatially organize the two polymeric matrices rather than simply blending them into a single layer. This configuration is expected to provide a more controlled diffusion pathway for the essential oil, thereby reducing its rapid migration and favoring sustained release. In addition, the distinct PVA and sodium alginate layers may contribute to balancing the mechanical and barrier properties of the films while limiting direct phase mixing between the two polymeric matrices. The incorporation of nanoclays can further increase the diffusion path and contribute to regulating the migration of the volatile compounds. (Ghayour and Ehtesabi, 2026; Miao et al., 2026).

To improve the physical and functional properties of polymeric films, one of the most widely adopted strategies is the incorporation of nanoclays. These inorganic mineral materials, whether natural, such as bentonite, or synthetic, such as layered double hydroxides (LDH), can be readily dispersed in various forms within polymer matrices. The resulting nanocomposite structure mainly depends on the type of clay used and the interactions established between the polymer matrix and the clay nanoparticles (Qu and Luo, 2021). Numerous studies have demonstrated that the combination of polymers and nanoclays can significantly enhance the mechanical, thermal, and barrier properties of packaging films (Kim et al., 2022; Romo-Uribe et al., 2026). However, the instability of essential oils within polymeric matrices, the limited performance of PVA-SA-based films, and the relatively high cost of several encapsulation technologies developed to date remain major challenges for food preservation applications.

Molecular docking is traditionally used to predict and investigate the interactions between bioactive compounds and biological targets, particularly proteins involved in microbial activity (Noshad et al., 2022). However, in active food-packaging studies, the interactions between essential oil constituents and polymeric matrices are often proposed based on indirect experimental evidence, such as FTIR, XRD, or changes in physicochemical properties. In this context, molecular docking can provide a complementary molecular-level approach for exploring the possible interactions between selected bioactive compounds and polymeric matrices (Rajendran et al., 2024). Such computational modeling may provide preliminary insights into the types of non-covalent interactions that can occur, including hydrogen bonding and van der Waals interactions, and can therefore complement experimental observations related to essential oil stabilization and retention within the polymer matrix. In the present study, docking was therefore employed as a complementary exploratory tool to investigate the possible interactions between selected constituents of Nigella sativa essential oil and the PVA and sodium alginate matrices, thereby providing molecular-level insights that support the interpretation of the experimental results.

Although our previous studies have explored chitosan and cellulose acetate-based films incorporating essential oils, the present work introduces a fundamentally different approach by developing a bilayer PVA/SA system reinforced with two complementary clay nanofillers (bentonite and LDH) and functionalized with Nigella sativa essential oil. We hypothesized that the incorporation of bentonite and LDH would enhance essential-oil retention and modulate its release within the PVA/SA bilayer matrix, while improving the barrier and functional properties of the films. Due to their different structural and surface characteristics, the two clays were expected to produce distinct effects on essential-oil release and, consequently, on the preservation performance of the resulting coatings.

Unlike previous studies that have generally investigated polymer/essential-oil systems or individual nanoclay-reinforced films, the present work provides a direct comparison of natural bentonite and synthetic LDH within the same PVA/SA bilayer platform (Punia Bangar et al., 2023). This approach allows their respective effects on essential-oil retention and release, film functionality, and preservation performance to be evaluated under comparable conditions. The combination of these material-level investigations with the application of the resulting formulations as coatings for refrigerated beef provides an integrated structure-release-preservation assessment.

In this context, the present study aims to investigate the effect of incorporating bentonite (BNT) and layered double hydroxide (LDH) on the physicochemical, mechanical, and functional properties of PVA-SA bilayer films, as well as their influence on the stability of Nigella sativa essential oil through controlled release analysis. Furthermore, the developed films were applied as spray coatings to evaluate their effectiveness in preserving refrigerated beef stored at 4 °C for 10 days using key freshness and quality indicators, including pH, color parameters, texture, total volatile basic nitrogen (TVB-N), thiobarbituric acid reactive substances (TBARS), and weight loss.

## 2 Materials and methods

### 2.1 Materials

Polyvinyl alcohol (PVA, degree of hydrolysis >99%) sodium alginate (SA, Low molecular weight), Tween 80, sodium hydroxide, hydrochloric acid, Trichloroacetic acid, Thiobarbituric acid, Boric acid, Magnesium chloride hexahydrate, Aluminum chloride nonahydrate, dimethyl sulfoxide (DMSO, 99%) and 2,2-diphenyl-1-picrylhydrazyl (DPPH) were purchased from Sigma-Aldrich. The *Nigella sativa* essential oil was extracted by clevenger hydrodistillation. Ethanol (99%) and acetic acid (99%) were supplied by the Solvachim laboratory (Casablanca, Morocco).

### 2.2 Preparation of LDHc

The Mg-AL LDH precursor was prepared by coprecipitation according to the method described in previous studies (El Meskine et al., 2025). Magnesium chloride hexahydrate (MgCl_2_·6H_2_O) and aluminum chloride nonahydrate (AlCl_3_·9H_2_O) were dissolved in distilled water with a molar ratio of Mg/Al = 3. The pH of the reaction mixture was maintained at 10 by the addition of a mixed solution of NaOH and Na_2_CO_3_. The resulting gel was stirred at room temperature for 24 h. The obtained clay was then washed by centrifugation with distilled water, dried at 60 °C for 24 h, ground, and sieved to retain particles smaller than 100 µm.

### 2.3 Characterization of clays

#### 2.3.1 Measurement of specific surface area by the BET method

The specific surface area was determined using the Brunauer–Emmett–Teller (BET) method with a MICROMERITICS ASAP 2020 accelerated surface area and porosimetry analyzer. The pristine bentonite and LDH samples were degassed under vacuum at 120 °C for 5 h prior to BET analysis. No thermal treatment or calcination was applied before the measurements. A moderate degassing temperature was selected to remove loosely retained moisture while minimizing potential structural modifications of the clay materials.

### 2.4 Characterization of *Nigella sativa* essential oil

#### 2.4.1 Chemical composition of NSEO

GC-MS analyses were performed using a Shimadzu GC-2010 gas chromatograph equipped with a BP-5 capillary column (30 m × 0.25 mm, 0.25 μm) and coupled to a Shimadzu QP2010 Plus mass spectrometer. The oven temperature program was identical to that used for GC. Helium was used as the carrier gas (36.5 cm s^-1^; split ratio 1:40). The transfer line and ion source were set to 300 °C and 200 °C, respectively. Mass spectra were recorded in EI mode at 70 eV over a scan range of 40–400 u. The identification of the compounds was carried out from the retention indices (n-alkanes in C9-C20) and by comparison of the mass spectra with commercial libraries (Shimadzu, NIST05) and an internal reference database (Elouaddari et al., 2019).

### 2.5 Preparation and characterization of *Nigella sativa* essential oil macroemulsion (EOM)

The EOM was prepared using tween 80 as a surfactant, according to the protocol described in other studies (Shen et al., 2022), with a few modifications. The emulsions were prepared by mixing Nigella sativa essential oil (10 % wt of PVA) and tween 80 (60 % wt of NSEO) at a ratio of 1:0.6 (V/V) in 5 mL of distilled water. After the formation of the essential oil micelles, natural clay (bentonite) or synthetic clay (LDH) was added at a 1:1 (w/w) ratio relative to the mass of essential oil. The mixture was then mechanically homogenized for 24 h. Microscopic analysis was carried out to visualise the micelles formed. The images obtained were analysed using ImageJ software (National Institutes of Health, Bethesda, MD, United States) to measure droplet diameter and particle size distribution. The zeta potential of the emulsifications formed was determined using a Malvern ZetaSizer Nano ZS90 instrument (UK). Three sets of measurements were performed, and the mean value and standard deviation were reported.

### 2.6 Preparation of bi-layer films

The PVA-SA bilayer films were prepared using the pouring method according to the protocol described in our previous studies (Bouftou et al., 2024b; 2024c). The PVA solution was first prepared by dissolving 1 g of PVA in 20 mL of distilled water with stirring at 80 °C for 24 h. Once completely dissolved, the PVA solution was poured into a Petri dish to form the first layer. The oil phase, previously prepared according to the protocol described in Section 2.5, was then carefully spread over the surface of the PVA layer. A film-forming sodium alginate (SA) solution was prepared separately by dissolving 0.2 g of SA in 10 mL of distilled water with stirring at 80 °C for 24 h. This SA solution was then poured over the PVA-oil layers to form the second layer. The poured solutions were left to dry at room temperature for 72 h. Once completely dry, the films were demolded and stored at room temperature until characterization.

### 2.7 Characterization of coating solutions

#### 2.7.1 Viscosity measurement

The viscosity of PVA-SA film solutions was tested using Anton Paar Rheometer, with a shear rate between 0 and 100 s^-1^ at room temperature. The tests for each film solution were carried out in triplicate.

#### 2.7.2 Interaction studies of major components of *Nigella sativa* essential oil and PVA-SA

To investigate the potential interactions between NSEO and the PVA-SA association, an *in silico* analysis was carried out. The dimensional structures of linalool, and Isopulegol were obtained from the PubChem database, while sodium alginate and poly (vinyl alcohol) (PVA) were constructed by modeling their respective repeating units, consisting of β-D-mannuronic and α-L-guluronic acid residues in the sodium salt form for alginate, and vinyl alcohol units bearing hydroxyl functional groups along the polymer backbone for PVA. All structures were imported into GaussView 6.0, where they were drawn and subjected to geometry optimization (*Clean 3D* followed by minimization using the MMFF94 force field) in order to obtain stable three-dimensional conformations. Molecular docking was then performed using AutoDock 4.0, and the predicted interactions were analyzed and visualized with BIOVIA Discovery Studio Visualizer.

### 2.8 Films characterization and properties

#### 2.8.1 Thickness and optical properties of PVA-SA bilayer films

The thicknesses of the films were measured, at ten random points of each label, using a micrometer (Mitutoyo Digimatic Micrometer Japan), with an accuracy of approximately 0.0001 mm. The thicknesses are expressed as average values of the ten measurements. And the opacity of the films was measured in accordance with the method reported by (Kanatt et al., 2012), using a spectrophotometer (UV5, METTLER TOLEDO), the films were cut in a rectangular fashion (1 × 3 cm^2^) and placed in the quartz cuvette of the apparatus, the absorbance of the films was recorded at 500 nm (A_500 nm_), the empty apparatus compartment is used as a reference.

Film opacity is measured using the following equation:

$$Opacity=\frac{A500\;nm}{X}$$ (1)

With A500 nm represents the light absorbance by the film at 500 nm and X is the thickness of the film (mm).

The color parameters (L*, a* and b*) of PVA-SA bilayer films were determined using a calibrated colorimeter (Datacolor 400™, Technical Color Solutions, Karachi, Pakistan) according to the method used by several previous studies (Bouftou et al., 2024a; Li et al., 2025). The color difference (ΔE) was then calculated using the following equation:

$$\Delta E=\sqrt{{\left(L*-L\right)}^{2}+{\left(a*-a\right)}^{2}+{\left(b*-b\right)}^{2}\;}$$ (2)

where L*, a*, and b* are the color values of the film sample; L = 70.37, a = 1.93, b = −3.25 are the values of the standard white board.

#### 2.8.2 Fourier-transform infrared spectroscopy

A Fourier transform infrared spectrophotometer (Jasco FT/IR-4600, Europe) was used to evaluate the chemical structure and composition of the developed PVA-SA films. Spectra were obtained in the wavelength range between 350 and 4,000 cm^-1^, with a resolution of 4 cm^-1^ and 32 cm^-1^.

#### 2.8.3 Scanning electron microscopy (SEM)

The morphological analysis of the surface and cross-section of the PVA-SA films were carried out by Thermo Scientific Apreo two scanning electron microscope (USA-Oregon), with a spatial resolution of 1.2 nm and under controlled atmosphere. The films were cut into pieces and fixed on stainless steel tubes before being analysed.

#### 2.8.4 X-ray diffraction (XRD)

The X-ray diffraction analysis of the PVA-SA films was performed by an X-ray diffractometer (D8 ADVANCE, BRUKER), characterised by a tube voltage of 40 KV and a current of 40 mA and a scanning speed of 0.5 s/step.

#### 2.8.5 Thermal properties

The thermal stability of the developed films was evaluated using differential scanning calorimetry (DSC) and thermogravimetric analysis (TGA). For DSC analysis, samples weighing approximately 4–5 mg were hermetically sealed in aluminum pans and placed in the instrument at room temperature. Measurements were carried out over a temperature range of 25 °C–270 °C at a heating rate of 10 °C/min under a nitrogen atmosphere with a purge gas flow rate of 50 mL/min. Thermogravimetric analysis (TGA) was performed using a DTG-60H (SHIMADZU, Japan). Samples weighing approximately 3.5–6.5 mg were placed in alumina crucibles and heated from 25 °C to 270 °C at a heating rate of 10 °C/min under an air atmosphere. The mass variation of the samples was continuously recorded as a function of temperature.

#### 2.8.6 Mechanical properties

The mechanical properties of the PVA-SA films, namely, tensile strength and elongation at break, were carried out using a mechanical testing machine (Hounsfield H50KS), with a 500 N load cell at room temperature. The displacement speed is 2 mm min^-1^ and at least five specimens were tested.

#### 2.8.7 Solubility in water and water absorption capacity

The solubility of PVA-SA films and their capacity to absorb water were determined by gravimetric method, using the protocol described in several studies, with a few modifications (Sobhan et al., 2019). Briefly, the PVA/CS films were cut into a rectangular format (30 mm*20 mm) and dried at a temperature of 40 °C for 1 h, and weighed (W_0_). The films were then placed in bottles containing 25 ml of distilled water and stored for 2 h at room temperature. The films were then dried using filter paper to remove surface water, and weighed (W_1_). Finally, the films were placed in the oven to dry, and weighed again (W_2_). The test was carried out in triplicate.

The solubility and water absorption of the films are calculated using the following equation:

$$Solubility\;\left(\%\right)=\frac{W0-W2}{W0}\times 100$$ (3)

$$Water\;absorption\;\left(\%\right)=\frac{W1-W0}{W0}\times 100$$ (4)

#### 2.8.8 Water contact angle (WCA)

Static contact angle measurements were performed at room temperature using a KRÜSS DSA25S droplet shape analyzer. Approximately 2 μL ultrapure water droplets were deposited in a controlled manner onto the sample surfaces using an automated dispensing system. Images of the droplets were captured by a high-resolution camera equipped with LED illumination and then processed with ADVANCE software, which determines the contact angle using the tangent circle method.

### 2.9 Functional characteristics of active films

#### 2.9.1 Ultraviolet and visible light transmission

The UV-visible light barrier properties of the films were evaluated using a UV-Vis spectrophotometer (METTLER TOLEDO), following the protocol described in our previous studies (Bouftou et al., 2024a; Islam et al., 2026). Rectangular specimens of bi-layer films with identical dimensions (10.0 cm × 1.5 cm) were cut and placed in the sample holder of the spectrophotometer, ensuring that the light beam directly impinged on the film surface. The transmittance spectra were recorded in triplicate over the wavelength range of 200–800 nm.

#### 2.9.2 Water vapor transmission rate (WVTR)

Water vapor transmission rate (WVTR) is one of the important criteria that food packaging needs to validate in order to extend the shelf life of foodstuffs and preserve their quality. WVTR of PVA-SA films was measured using the method described by (Boonkaew et al., 2014), with modifications. The opening of a 2.5 cm diameter bottle, filled with 10 ml of distilled water, was hermetically sealed with elaborate films and weighed (W_0_). The bottles were placed in the oven for 24 h at a temperature of 40 °C and weighed again (W_1_). The WVTR of the films was calculated using the following equation:

$$WVTR=\frac{W0-W1}{A\times 24}\times 10^{6}$$ (5)

#### 2.9.3 Antioxydant activity

To study the antioxidant activity of the prepared PVA-SA films, we adapted the DPPH method, according to the protocol described by Gomaa F. et al. with some modifications (El Fawal et al., 2019). The DPPH solution is prepared by dissolving 6 mg of DPPH in 50 ml of methanol, at the same time a sample of the 50 mg of our film is immersed in 5 ml of ethanol. The two mixtures are collected in a test tube. After 30 min of incubation, we measured the absorbance at 517 nm using a spectrophotometer (UV5, Mettler-Toledo). The percentage of free radical scavenging activity is measured using the following equation:

$$\left(\%\right)\;Antiradical\;activity=\frac{Abs\;control-Abs\;sample}{Abs\;control}\times 100$$ (6)

With Abs control is the absorbance of DPPH solution without extract and Abs sample the absorbance of DPPH solution in the presence of extract.

#### 2.9.4 Antibacterial activity

The *in vitro* antibacterial activity of the investigated films was performed against the following pathogenic bacteria: *S. aureus* (Gram-positive bacteria) and *E. coli*, (Gram-negative bacteria). The strains were sub-cultivated on NA (Nutrient Agar) solid medium at 37 °C. The antibacterial activities of the PVA/CS films were determined using the agar disc diffusion method as described by Bauer (Bauer and Apostolov, 1966). Bacteria that were grown overnight were diluted to a concentration of 3 × 108 CFU mL^-1^. Films discs with 6 mm diameter were placed on either Muller-Hinton solid medium previously amended with 3 × 108 CFU mL^-1^ of bacteria. The inoculated plates were incubated at 37 °C for 24 h. The antibacterial activity was evaluated by measuring the clearance zone around the discs, which indicates the positive antibacterial activity of the products. Each experiment was repeated 3 times.

#### 2.9.5 Cumulative release behavior in food simulants

The measurement of the Cumulative release of NSEO in PVA-SA bilayer films was determined according to the protocol described by (Zhang T. et al., 2022) with some modifications. Briefly, the rate of release of NSEO was assessed in DMSO. Film samples (50 mg) were fully immersed in flasks containing 20 mL of DMSO and stored in the dark at room temperature for 30 days. Prior to the release study, a preliminary UV–Vis screening was performed by dissolving the same amount of NSEO incorporated into the films in DMSO. The maximum absorbance was experimentally observed at 290 nm; therefore, this wavelength was selected to monitor the release of NSEO from the films. Every 2 days, the absorbance of the solutions was measured at 290 nm using a UV–Vis spectrophotometer. The cumulative release of NSEO was then determined according to the following equation:

$$Cumulative\;release\;\left(\%\right)=\frac{Abs\;quantity\;released}{Abs\;quantity\;added}\times 100$$ (7)

### 2.10 Preservation test

The coating solutions corresponding to the films exhibiting the best functional properties were subsequently applied to beef fillets by spraying, using the same formulation with identical composition, component ratios, applied amount, and drying time as those used for the cast films, thereby ensuring comparable film formation on petri dishes and on the beef surface.

#### 2.10.1 Contact angle on beef surface

The static contact angle of the coating formulations on the beef surface was measured at room temperature using a KRÜSS DSA25S drop shape analyzer. A small droplet of the formulation (∼5 μL) was gently deposited on the cleaned and slightly dried surface of the beef fillets using a micropipette. The droplet image was captured immediately by a high-resolution camera with LED illumination. The contact angle was calculated using the tangent circle method with the ADVANCE software. Each measurement was performed in triplicate at different locations on the fillet to ensure reproducibility (Fettah et al., 2025).

#### 2.10.2 Colour measurement

The color of the fillet surface was measured directly using a Minolta CR-400 chroma meter (Konica Minolta Inc., Osaka, Japan) without removing the coating, as this corresponds to the color perceived by consumers. The instrument, equipped with a CR-400 measuring head, was calibrated against a standard white calibration plate prior to use. Measurements were performed by placing the measuring head on the surface of the beef fillet samples, and the CIE color parameters L* (lightness), a* (redness), and b* (yellowness) were recorded three times at different points for each sample (Liu et al., 2020).

#### 2.10.3 Weight loss

The percentage of weight loss of beef samples was determined according to the protocol described in the literature (Mohlamonyane et al., 2026). At the beginning of the storage period, each beef sample packaged with a given formulation was weighed using an electronic balance, and the initial weight (Wi) was recorded. The same samples were then weighed every 2 days to determine the final weight (Wf). The weight loss was subsequently calculated using the following equation:

$$Weight\;loss\;\left(\%\right)=\frac{Wi-Wf}{Wi}\times 100$$ (8)

#### 2.10.4 pH measurement

1 g of each ground beef sample was homogenized using an electric blender. The resulting homogenate was mixed with 100 mL of distilled water, stirred for 5 min, and subsequently filtered through filter paper. Finally, the pH of the filtrate was measured using a calibrated pH meter (METTLER TOLEDO) (Cai et al., 2022).

#### 2.10.5 Determination of lipid oxidation

Lipid oxidation in beef fillets was evaluated using the thiobarbituric acid reactive substances (TBARS) method, adapted from previously reported protocols with minor modifications (Cai et al., 2021). 2.5 g of each sample were homogenized with 25 mL of 7.5% trichloroacetic acid (TCA) solution for 2 min, and the mixture was then filtered. An aliquot of 5 mL of the filtrate was mixed with 5 mL of 0.02 M thiobarbituric acid (TBA) solution and manually stirred until homogenized. The mixture was subsequently heated in a water bath at 100 °C for 30 min, resulting in the development of a light pink color, indicating the expected chemical reaction. After cooling in an ice bath, the absorbance of the supernatant was measured at 532 nm using a spectrophotometer (UV5, METTLER TOLEDO). The TBARS values were calculated according to the following equation:

$$TBARS\;\left(mg\frac{MDA}{Kg}\right)=\frac{A}{M}x\;9.48$$ (9)

where A was the absorbance value at 532 nm, M was 2.5 g.

#### 2.10.6 Total volatile basic nitrogen (TVB-N)

The total volatile basic nitrogen (TVB-N) content was determined by steam distillation according to the method described in the literature, with some modifications (Tshamisane et al., 2025). Beef samples (0.5 g) were homogenized with 25 mL of distilled water, and the mixture was stirred for 30 min at room temperature using a magnetic stirrer. The mixture was then filtered through filter paper. The obtained filtrate was alkalized by adding 2.5 mL of NaOH solution (10 g/L) and subsequently subjected to distillation for 5 min. The distillate was collected in a flask containing 10 mL of 2% boric acid along with a few drops of bromocresol green as a color indicator. Finally, the mixture was titrated with 0.01 mol/L hydrochloric acid (HCl) solution. The TVB-N content was calculated according to the following equation:

$$TVB-N\;\left(\frac{mg}{100\;g}\right)=\frac{\left(V1-V2\right)\;x\;C\;x\;14}{m\;x\;10/100}$$ (10)

Where V_1_ and V_2_ represent the titration equivalence volumes of the sample and the blank, respectively; C is the molar concentration of the HCl titrant solution (mol/L); and m is the mass of the beef sample (g).

#### 2.10.7 Statistical analysis

The experiments were conducted three times using a fully randomized design with a factorial scheme. Subsequently, the collected data underwent an analysis of variance (ANOVA), followed by either Tukey’s test or linear regression at a 5% probability level for further examination. All statistical procedures were carried out using the SAS software (SAS Institute Inc., Cary, NC, USA).

## 3 Results and discussion

### 3.1 Measurement of specific surface area by the BET method

The BET specific surface area results reveal a significant difference between the two materials (p < 0.05) (Table 1). The natural bentonite exhibits a relatively low surface area (23.299 m^2^/g), which is typical for raw clays due to partial pore blockage by impurities and limited accessible porosity (Alexander et al., 2018). In contrast, the LDH-Mg_3_-Al-CO_3_ sample shows a markedly higher surface area (79.7918 m^2^/g), approximately 3.4 times greater than that of bentonite. This increase can be attributed to the lamellar structure of layered double hydroxides, which provides a higher degree of porosity and a larger number of accessible active sites. The enhanced surface area of LDH-Mg_3_-Al-CO_3_ suggests improved adsorption capacity and greater reactivity, making it more suitable for applications such as adsorption, catalysis, and pollutant removal compared to raw bentonite.

**TABLE 1:** Compositions and weight percentages of the components in the prepared films.

Encapsulation methods	Films	PVA	SA	Bentonite	LDH	NSEO	Tween 80
Blanc 1	PVA	**1 g**	**-**	**-**	**-**	**-**	**-**
Blanc 2	SA	-	0.2 g	-	-	-	-
Blanc 3	PVA-SA	1 g	0.2 g	-	**-**	**-**	**-**
Direct incorporation	PVA-SA-EO	1 g	0.2 g	**-**	**-**	0.1 g (10%)	0.06 g
Natural clay	PVA-SA-EO-BNT	1 g	0.2 g	0.1 g (10%)	-	0.1 g (10%)	0.06 g
Synthetic clay	PVA-SA-EO-LDH	1 g	0.2 g	-	0.1 g (10%)	0.1 g (10%)	0.06 g

### 3.2 Chemical composition of NSEO

The results of the gas chromatography-mass spectrometry (GC-MS) analysis of Nigella sativa essential oil, obtained by clevenger hydrodistillation extraction from Nigella sativa seeds, are presented in Table 2. The chemical composition of the oil, expressed as relative percentage and retention time of the major identified compounds, is summarized therein. The results revealed that the essential oil is composed of a total of 30 different compounds. These findings are consistent with those reported in the literature regarding the chemical composition of Nigella sativa essential oil (Rasul et al., 2022). The major constituents identified mainly belong to monoterpene hydrocarbons, oxygenated monoterpenes, sesquiterpenes, as well as aromatic and phenolic compounds, as detailed below.

**TABLE 2:** Specific surface area of bentonite and LDH clays.

Clays	S_BET_ (m^2^/g)
Bentonite	23.299
LDH-Mg_3_-Al-CO_3_	79.7918

### 3.3 Microstructural analysis, zeta potential, particle size, and particle size distribution index

The particle size and surface charge of micelles play a crucial role in determining their colloidal stability (He et al., 2024). The results of the average particle size and zeta potential measurements are presented in Figures 1a–c. The increase in particle size from nanoscale micelles (0.184 µm) to micrometer-sized structures (2.353–2.420 µm) in the presence of bentonite and LDH can be attributed to the formation of micelle-clay aggregates through adsorption and bridging effects. The interaction between essential oil micelles and clay particles reduces colloidal stability, promoting flocculation and the formation of larger structures (Yang et al., 2023). Despite their different surface charges, both bentonite and LDH exhibit similar aggregation behavior, resulting in comparable particle sizes.

**FIGURE 1:**
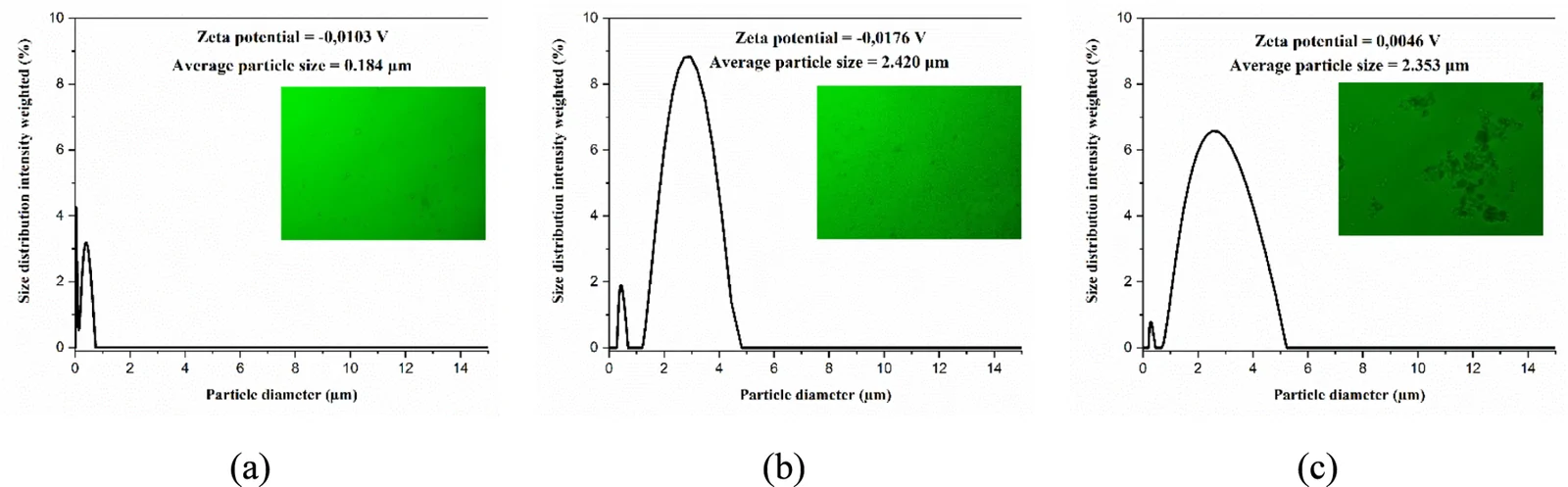
Microscope images of emulsions, particle size distribution and zeta potential of **(a)** essential oil micelles; **(b)** essential oil micelles-bentonite; **(c)** essential oil micelles-LDH.

The zeta potential values of the systems were relatively low (−10.3 mV for micelles, −17.6 mV for micelles with bentonite, and +4.6 mV for micelles with LDH), indicating limited colloidal stability. The increase in negative charge in the presence of bentonite suggests slight stabilization; however, the values remain insufficient to prevent aggregation. In contrast, the near-neutral charge observed in the LDH system indicates charge neutralization, which promotes strong flocculation. These findings explain the significant increase in particle size observed in both systems, confirming that aggregation is mainly governed by reduced electrostatic repulsion and enhanced particle-particle interactions (Yang et al., 2023; He et al., 2024).

### 3.4 Characterization of coating solutions

#### 3.4.1 Viscosity measurement

A rheological study was carried out on the polymer solutions prior to film preparation in order to determine the most appropriate preparation method to adopt. The viscosity results as a function of shear rate are presented in Figure 2a. These results showed that all solutions behave as non-Newtonian fluids, more specifically pseudoplastic fluids, characterized by a decrease in viscosity with increasing shear rate (Bouftou et al., 2024b; 2026b; Lamine and Hifdi, 2026). This pseudoplastic behavior is advantageous for spray application because the decrease in viscosity at higher shear rates facilitates the flow and atomization of the coating solution during spraying, while the higher viscosity under lower shear conditions can favor retention of the coating on the beef surface and contribute to film formation after deposition. Therefore, the observed rheological behavior provides a suitable balance between processability during spraying and sufficient viscosity for coating formation and retention (Wang et al., 2022). Furthermore, the PVA-SA blend exhibits a higher viscosity compared to the individual polymers. This increase is attributed to strong intermolecular hydrogen-bonding interactions (Figure 2b), leading to the formation of a physically entangled network (characterized by FTIR and XRD afterwards) (Zhang T. et al., 2022; Lamine and Hifdi, 2025; Madihalli et al., 2025). In light of these results, particularly the high viscosity and the interactions between the polymers, the choice was made to prepare the films as bilayer systems, in order to overcome issues related to miscibility and processing associated with the blend.

**FIGURE 2:**
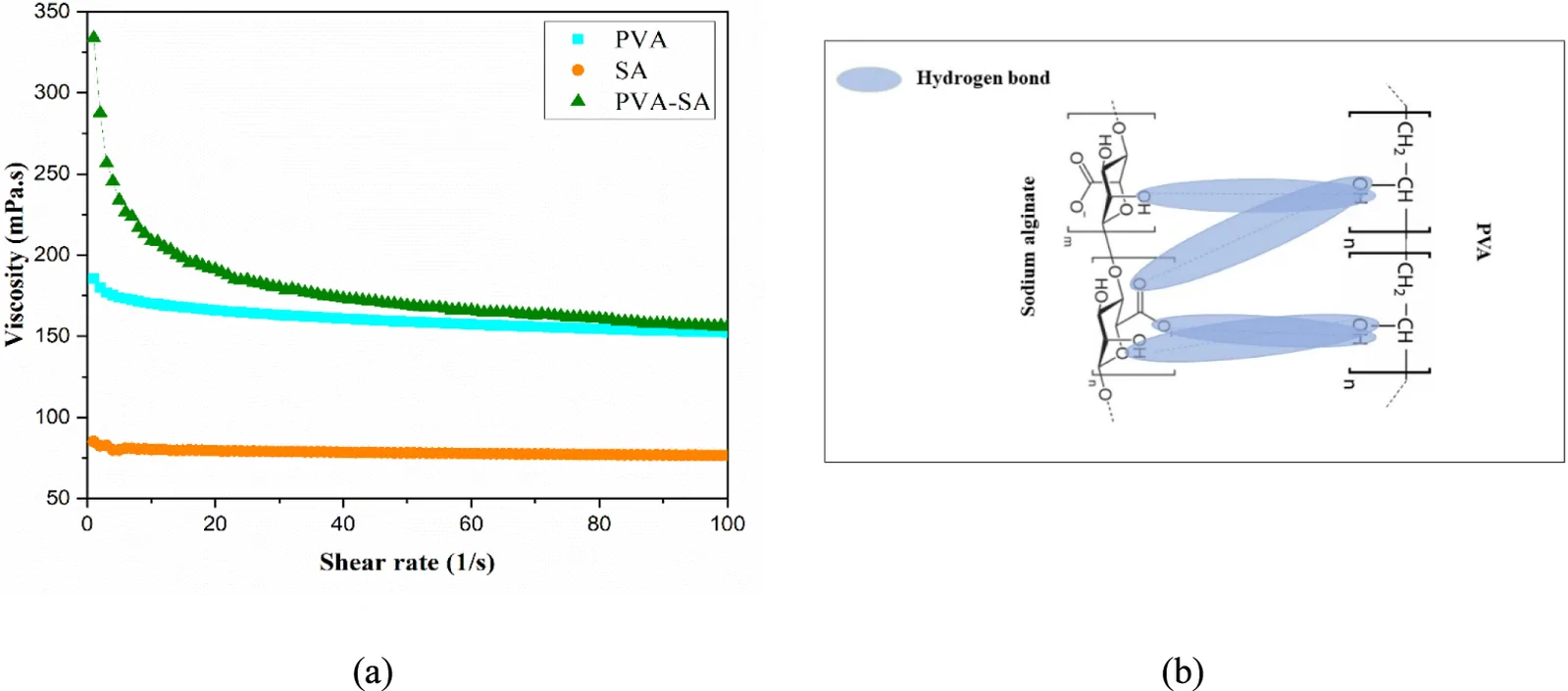
Viscosity of film-forming solutions of PVA, SA, and PVA–SA **(a)** and intermolecular interactions between PVA and sodium alginate **(b)**.

#### 3.4.2 Interaction studies of major components of *Nigella sativa* essential oil and PVA-SA

Molecular docking studies were carried out to evaluate the interactions and affinity between two constituents of *Nigella sativa* essential oil, namely, linalool and Isopulegol, and the bilayer polymeric matrix based on PVA and sodium alginate (Figure 3). The obtained binding energy values are presented in Table 3. The results showed that the binding energies ranged between −1.6 and −1.9 kcal/mol. These negative values indicate that the interactions are thermodynamically favorable (Bouftou et al., 2026b). The relatively low binding energy values can be attributed to the small size of the volatile molecules, the hydrophilic nature of both polymeric matrices, as well as the type of interactions involved, which are mainly hydrogen bonding and van der Waals interactions (Rajendran et al., 2024). From a comparative perspective, linalool exhibited nearly similar interaction energies with both PVA and sodium alginate, whereas isopulegol showed a slightly higher affinity toward PVA compared to sodium alginate. These findings suggest that the essential oil compounds of *Nigella sativa* are physically adsorbed within the polymer chains. Moreover, the weak nature of these interactions represents an advantage for the development of active films, as it facilitates the controlled release of bioactive compounds while preserving their functional properties.

**FIGURE 3:**
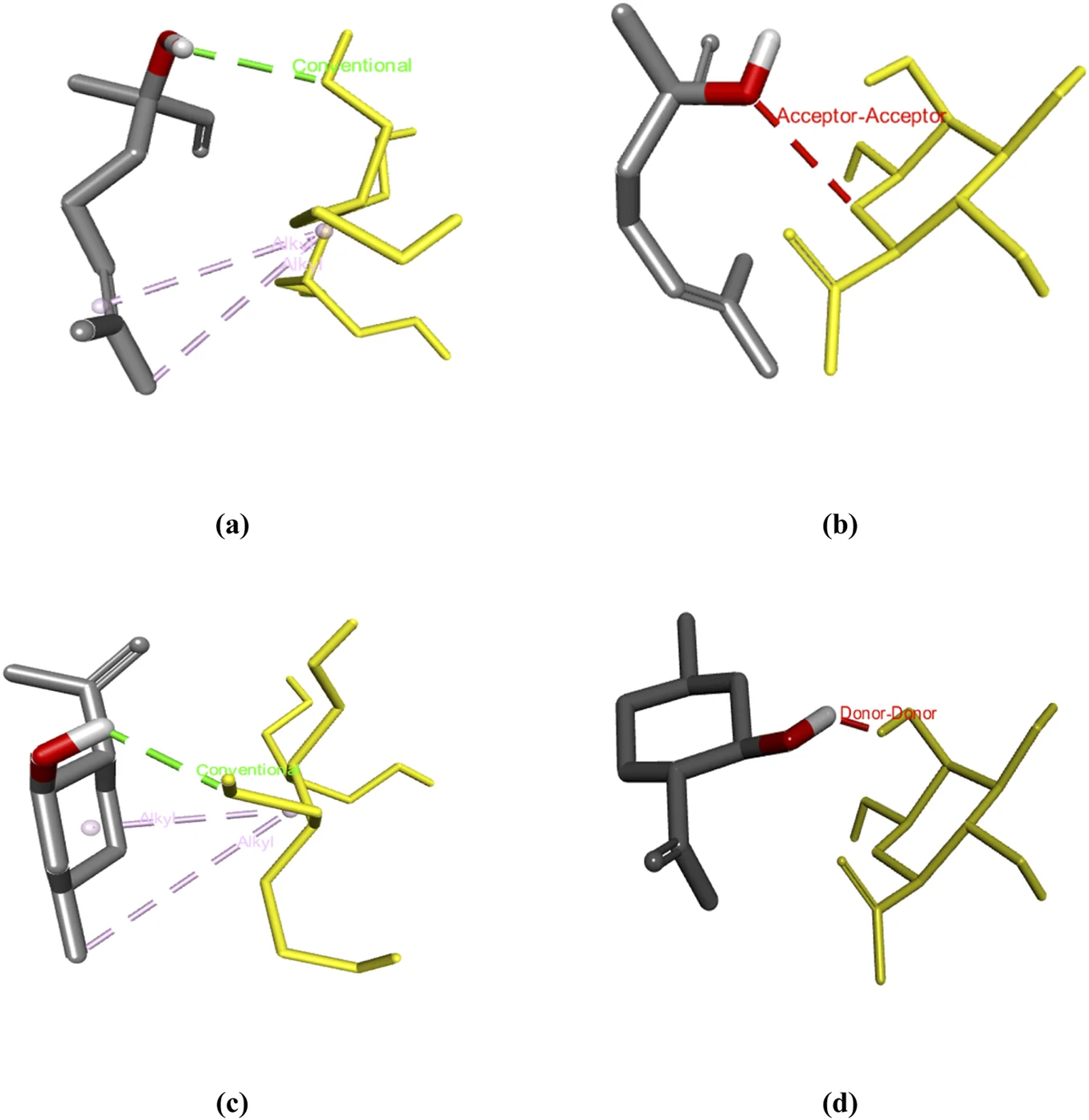
Comparative analysis of the binding energies of Linalool with **(a)** PVA and **(b)** SA, and between Isopulegol with **(c)** PVA and **(d)** SA.

**TABLE 3:** Major chemical constituents of NSEO.

N°	Compound	RT	%
1	Tricyclo [2.2.1.0 (2,6)]heptane, 1,3,3-triméthyl-	5.03	7.28**%**
2	Bicyclo [3.1.1]heptane, 6,6-diméthyl-2-méthylène-	5.73	2.71%
3	à-Phellandrene	6.17	4.69%
4	p-Cymene	6.52	1.37%
5	Linalool	7.73	1.14%
6	Isopulegol	9.06	1.69%
7	Bioallethrin	12.42	4.69%
8	Bicyclo [3.1.1]hept-2-ene-2-ethanol, 6,6-dimethyl-	12.90	6.43%
9	Benzene, 1-methyl-3,5-bis(1-methylethyl)-	13.69	6.36%
10	Benzene, 1-(1,1-dimethylethyl)-3,5-dimethyl-	13.89	17.74%
11	Benzene, 1,3,5-tris(1-methylethyl)-	14.11	1.09%
12	Benzene, 1,3-diethyl-	14.56	9.39%
13	Butylated Hydroxytoluene	14.78	2.73%
14	3-Cyclohexene-1-carboxaldehyde, 1,3,4-trimethyl-	14.85	2.94%
15	Thymol	21.66	0.64%
16	Carvacrol	22.19	8.85%

### 3.5 Films characterization and properties

#### 3.5.1 Thickness and optical properties

The thickness measurement results of the films, presented in Table 4, show that the control films based on sodium alginate exhibit lower thickness compared to those based on PVA. This difference can be attributed to the variation in the quantities used during the preparation of the bilayer films (Table 5). Moreover, the PVA-SA bilayer films exhibit higher thickness, which can be explained by the combination of the two polymeric layers. The incorporation of *Nigella sativa* essential oil (EO), in the form of micelles, did not lead to any statistically significant change in film thickness (p > 0.05). This behavior is consistent with the surface scanning electron microscopy (SEM) observations (Figure 7), which indicate a good dispersion of the essential oil micelles within the PVA-SA polymeric matrix. Its micrograph reveals the presence of a few non-dispersed droplets; however, the overall surface remains predominantly homogeneous (Figure 4). These results are consistent with several literature studies indicating that the effect of essential oils on film thickness depends on their nature, chemical composition, as well as their interaction and compatibility with the polymeric matrix (Hanani, 2016). In contrast, the incorporation of essential oil micelles in the presence of bentonite or LDH resulted in a significant increase in film thickness (p < 0.05). However, no significant difference was observed between films loaded with bentonite and those containing LDH (p > 0.05). This increase can be attributed, on the one hand, to the higher solid content per unit surface area, and on the other hand, to the enhanced interactions between PVA, SA, and the essential oil, promoted by the presence of clay fillers (Beigzadeh Ghelejlu et al., 2016). A similar behavior has been reported in a recent study highlighting the effect of montmorillonite and ginger essential oil on the increased thickness of chitosan-based films (Souza et al., 2019).

**TABLE 4:** Interaction energies between molecules Linalool and Isopulegol and matrices PVA and SA.

Molecule	PVA	SA
Linalool	−1.7 kcal/mol	−1.6 kcal/mol
Isopulegol	−1.9 kcal/mol	−1.6 kcal/mol

**TABLE 5:** Physical and optical properties (thickness, L, a, b*, ΔE, and opacity) of PVA-SA bilayer films.

Films	Thickness (mm)	L*	a*	b*	ΔE	Opacity
PVA	0.143 ± 0.004^b^	94.51 ± 1.12^d^	−0.98 ± 0.82^b^	5.02 ± 1.16^a^	25.68 ± 0.76^b^	0.07 ± 0.08^a^
SA	0.063 ± 0.012^a^	90.85 ± 0.85^c^	−0.25 ± 0.12^e^	15.23 ± 0.34^d^	27.67 ± 0.45^c^	21.49 ± 0.10^f^
PVA-SA	0.207 ± 0.017^c^	93.19 ± 0.14^d^	−1.06 ± 0.22^a^	9.51 ± 0.26^b^	26.31 ± 0.52^b^	3.71 ± 0.14^b^
PVA-SA-EO	0.214 ± 0.008^c^	91.43 ± 0.10^c^	−0.78 ± 0.34^c^	11.74 ± 0.17^c^	25.99 ± 0.22^b^	6.22 ± 0.18^d^
PVA-SA-EO-BNT	0.342 ± 0.016^d^	77.34 ± 0.24^a^	−0.47 ± 0.16^d^	15.41 ± 0.23^d^	20.06 ± 0.15^a^	6.79 ± 0.12^e^
PVA-SA-EO-LDH	0.339 ± 0.014^d^	89.18 ± 0.34^b^	−0.80 ± 0.28^c^	19.26 ± 0.11^e^	29.46 ± 0.18^d^	5.07 ± 0.21^c^

**FIGURE 4:**
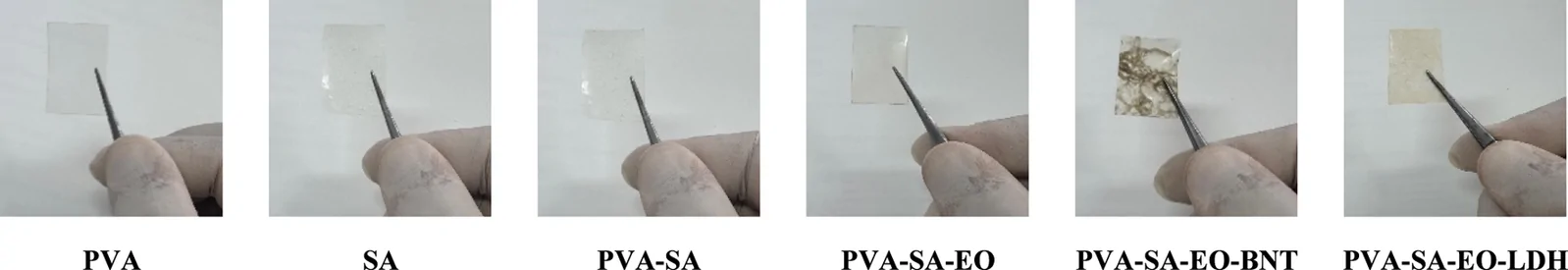
Visual morphology of PVA–SA bilayer films The different letters indicate that the samples are of significant difference (p < 0.05).

Table 5 presents the opacity and colorimetric parameters (L*, a*, b*, and ΔE) of the prepared films, highlighting the relationship between optical clarity and color variation. The PVA films exhibited a very low opacity (0.07 ± 0.08), indicating high transparency, along with a ΔE value of 25.68 ± 0.76, reflecting a noticeable but moderate color difference. In contrast, sodium alginate (SA) films showed a significantly higher opacity (21.49 ± 0.10) and a higher ΔE (27.67 ± 0.45), suggesting that their more turbid structure is associated with greater light scattering and color deviation. The PVA–SA bilayer films displayed intermediate opacity (3.71 ± 0.14) and ΔE (26.31 ± 0.52), indicating that the combination of the two polymers leads to a balanced optical behavior. This result is consistent with thickness measurements and suggests partial compatibility between the two layers, allowing moderate light transmission while maintaining a relatively uniform color. The incorporation of *Nigella sativa* essential oil (EO) micelles resulted in an increase in opacity (6.22 ± 0.18) and a slight decrease in ΔE (25.99 ± 0.22). This behavior suggests that, although light transmission is reduced due to increased scattering (likely caused by micellar structures and reduced interchain spacing) (Adilah et al., 2018), the color distribution becomes slightly more homogeneous. This observation is supported by SEM analysis, where the dispersion of micelles within the polymer matrix limits large-scale heterogeneities. Similar trends have been reported in films incorporating plant extracts or essential oils, such as *Chrysanthemum* extract (Madihalli et al., 2025) and chitosan-based films containing cinnamon (Perdones et al., 2014) or thyme essential oils (Peng and Li, 2014). In the presence of clay fillers, both opacity and colorimetric properties were significantly affected. The PVA-SA-EO-BNT films exhibited increased opacity (6.79 ± 0.12) and a marked decrease in ΔE (20.06 ± 0.15), indicating that bentonite enhances light scattering while promoting a more uniform color distribution, likely due to improved dispersion and strong polymer–filler interactions. Conversely, PVA-SA-EO-LDH films showed lower opacity (5.07 ± 0.21) but a higher ΔE (29.46 ± 0.18), suggesting reduced light scattering but greater color heterogeneity, possibly due to differences in particle dispersion and interaction mechanisms. Overall, these results demonstrate that both opacity and ΔE are strongly influenced by the film composition, particularly the presence of essential oil micelles and clay fillers. Their combined analysis provides deeper insight into the structural organization, dispersion state, and intermolecular interactions within the polymeric matrix.

#### 3.5.2 Water contact angle (WCA), solubility in water and water absorption capacity

Water contact angle (WCA) measurement is a key technique for evaluating the surface hydrophobicity or hydrophilicity of polymeric films, which is a crucial parameter for food packaging applications. A low contact angle (<65°) indicates a hydrophilic surface, whereas higher values reflect a hydrophobic character, enabling reduced moisture adsorption and improved water barrier performance.

As shown in Figure 5a, pure PVA and SA films exhibit low contact angle values of 33.1° and 38.3°, respectively, confirming their strongly hydrophilic nature due to the abundance of hydroxyl groups (Sonker et al., 2017; Shi et al., 2024). The combination of both polymers in the PVA-SA bilayer film leads to a notable increase in WCA to 56.3°, indicating a partial reduction in surface hydrophilicity resulting from intermolecular interactions between the polymer chains. The incorporation of essential oil into the bilayer matrix (PVA-SA-EO) causes a pronounced increase in the contact angle to 74.3°, demonstrating a significant enhancement in surface hydrophobicity (p < 0.05). These results clearly indicate that the essential oil effectively converts the initially hydrophilic PVA-SA bilayer films into hydrophobic systems, which is highly desirable for food packaging applications (Soleiman-Dehkordi et al., 2024). This behavior is attributed to the presence of hydrophobic compounds in the essential oil that reduce the availability of polar groups at the film surface. In contrast, the addition of bentonite to the system (PVA-SA-EO-BNT) results in a decrease in WCA to 53.8°, suggesting an increased affinity for water. This effect is likely related to the hydrophilic nature of bentonite and the exposure of hydroxyl groups at the film surface. Conversely, films containing layered double hydroxides (PVA-SA-EO-LDH) exhibit a relatively high contact angle of 69.2°, indicating that LDH not only preserves but also stabilizes the hydrophobicity induced by the essential oil. This behavior can be attributed to improved compatibility and dispersion of LDH within the PVA-SA bilayer matrix. Overall, these findings demonstrate that water contact angle analysis is an essential tool for correlating film composition with surface properties. The essential oil plays a pivotal role in transforming PVA-SA bilayer films from hydrophilic to hydrophobic systems, while LDH incorporation specifically contributes to maintaining and optimizing this hydrophobic character. As a result, these films exhibit surface properties that are particularly suitable for food packaging applications where reduced moisture uptake and enhanced food stability are required.

**FIGURE 5:**
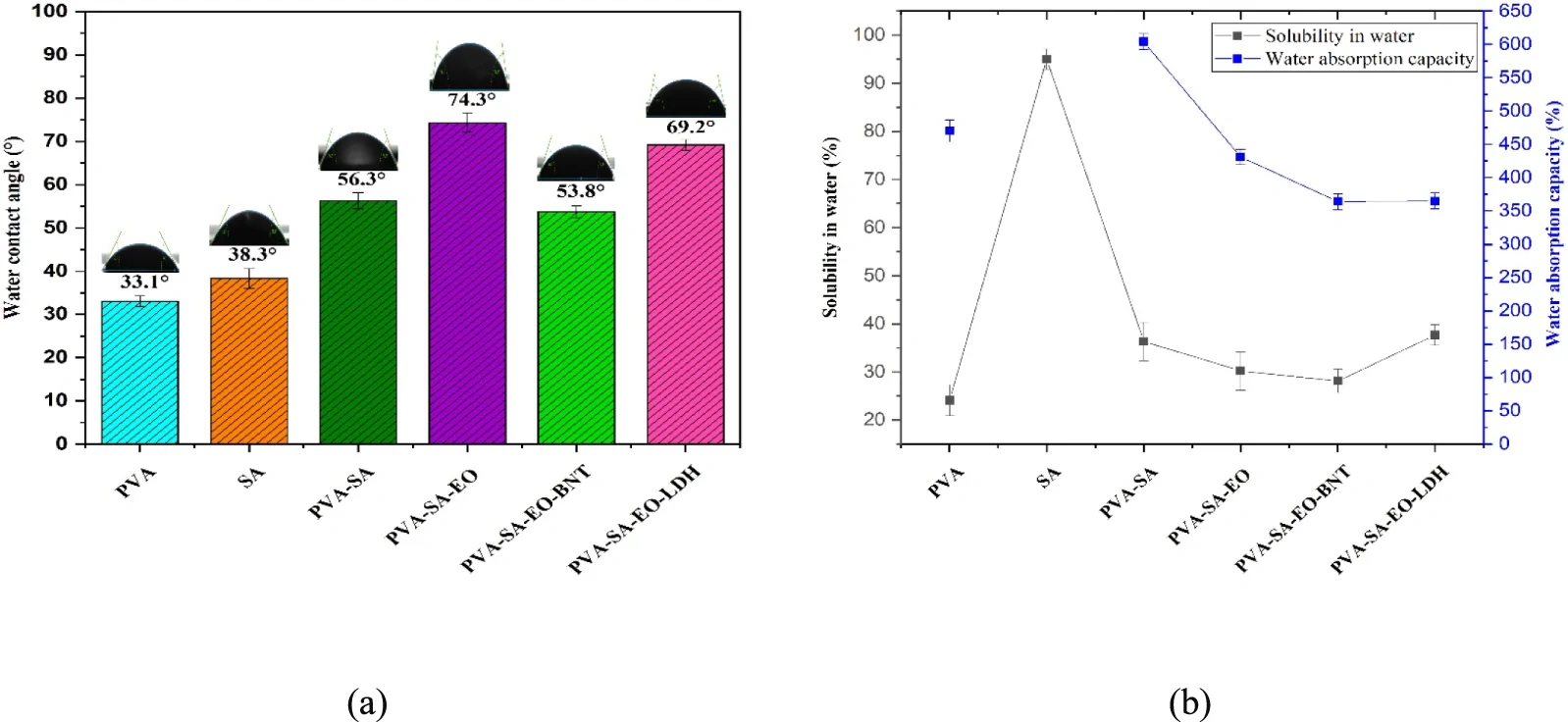
Water contact angle **(a)** and water solubility and water absorption capacity **(b)** of PVA-SA bi-layer films.

Water solubility and moisture absorption capacity are key parameters for evaluating the performance of food packaging materials. Generally, films with low water solubility and reduced moisture uptake are more suitable for packaging applications (Madihalli et al., 2025). Figure 5b presents the water solubility and moisture absorption results of the developed films. The results showed that PVA films exhibited moderate water solubility (24.12%) and relatively high moisture absorption capacity, which can be attributed to the presence of hydrophilic hydroxyl groups, despite their semi-crystalline structure confirmed by XRD analysis (Figure 8). In contrast, sodium alginate (SA) films displayed extremely high solubility (95.01%) due to their strong affinity for water, which prevented the measurement of their moisture absorption capacity. The incorporation of SA into PVA led to an increase in both water solubility (36.34%) and moisture absorption capacity of the bilayer films compared to neat PVA. This behavior is mainly related to the highly hydrophilic nature of SA and the reduction in crystallinity of the PVA matrix, as confirmed by XRD analysis (Figure 8), facilitating water diffusion within the film structure. The addition of essential oil resulted in a decrease in both water solubility and moisture absorption capacity of the PVA–SA bilayer films (p < 0.05). This reduction can be attributed to the formation of intermolecular interactions, particularly hydrogen bonding between the bioactive compounds and the polymer matrix, which reduces the availability of hydrophilic groups and limits water penetration (Zhai et al., 2017). Furthermore, the incorporation of clay fillers (bentonite and LDH) led to a further reduction in both solubility and moisture absorption capacity. This effect is more pronounced for bentonite due to its layered structure, which creates a barrier effect, enhances film compactness, and restricts water diffusion (Souza et al., 2019). In comparison, LDH also reduced water uptake but to a lesser extent, likely due to its relatively more hydrophilic nature (El Meskine et al., 2025).

#### 3.5.3 Fourier-transform infrared spectroscopy

To evaluate the effect of different additives on the chemical structure of the prepared films, Fourier-transform infrared (FTIR) spectroscopy was performed. The FTIR spectra of bentonite clay and LDH, illustrated in Figure 6a, revealed that the FTIR spectrum of bentonite clay exhibits an absorption band at 3,618.2 cm^-1^, corresponding to the stretching vibrations of structural hydroxyl groups characteristic of bentonite. The band observed at 1,636.4 cm^-1^ is attributed to the bending vibrations of OH groups of water adsorbed between the layers (Madejová, 2003). The Si-O stretching vibrations are responsible for the most intense absorption band, located at 1,008.4 cm^-1^ (Hebbar et al., 2018). Furthermore, the band at 915.3 cm^-1^ is associated with Al-Al-OH groups, while those at 529.8 and 457.4 cm^-1^ are attributed to Al-O-Si and Si-O-Si bending vibrations, respectively (Elashery et al., 2023). The FTIR spectrum of LDH highlights the characteristic bands typical of this type of material. A broad band centered at 3,453.5 cm^-1^ is attributed to the stretching vibrations of hydroxyl groups ν(OH). The band located around 1,637.8 cm^-1^ corresponds to the bending vibration of interlayer water δ(H_2_O). Furthermore, the presence of an intense band at 1,380.3 cm^-1^ is characteristic of the ν_3_ stretching mode of carbonate ions (CO_3_^2-^) intercalated within the interlayer spaces (El Meskine et al., 2025). Finally, the region between 400 and 800 cm^-1^ is associated with the vibrations of metal–oxygen and metal–hydroxyl bonds (M–O and M–OH), where M refers to the metal cations constituting the brucite-like layers (Lazaridis and Asouhidou, 2003).

**FIGURE 6:**
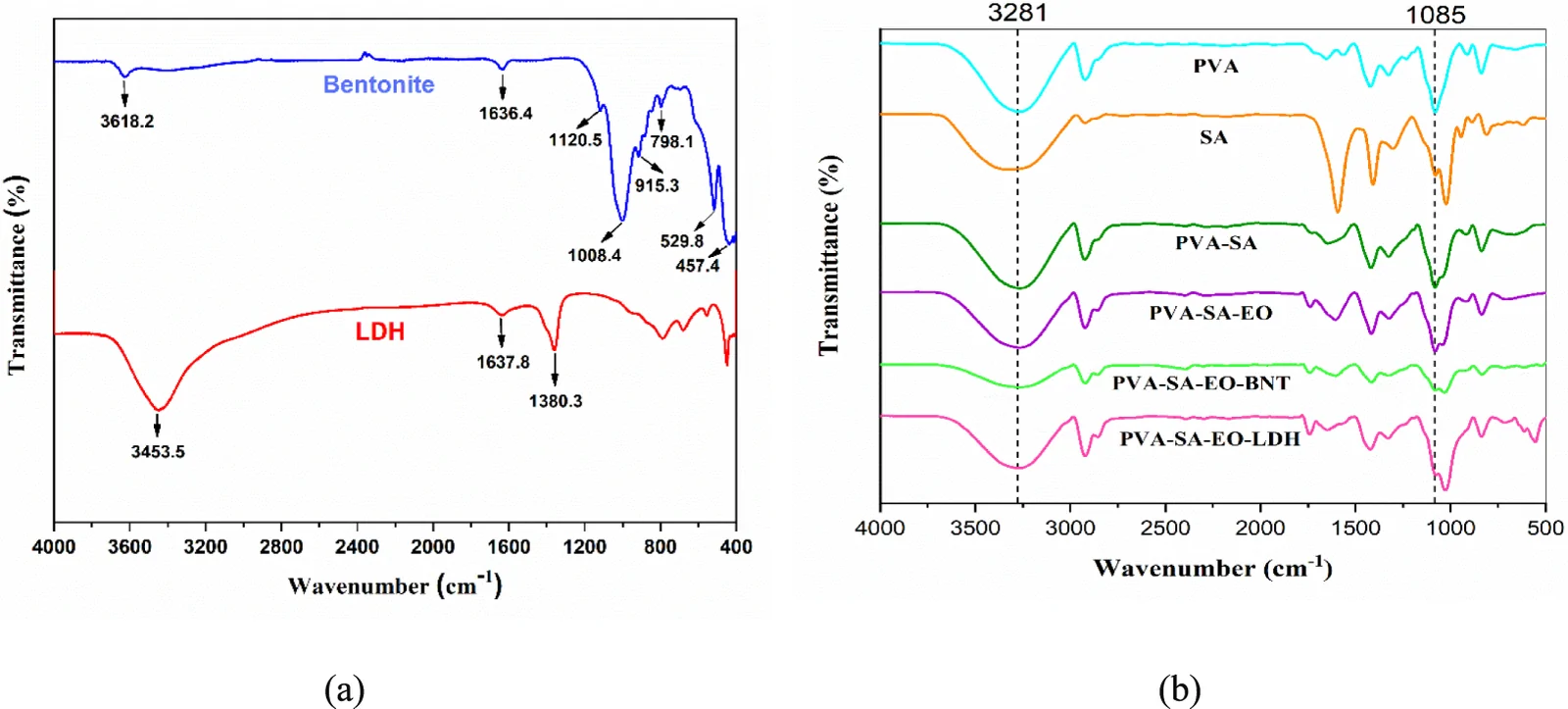
FT-IR spectra of the clays **(a)** and PVA-SA bi-layer films **(b)**.

The FTIR spectra of the prepared PVA–SA bilayer films are presented in Figure 6b. The characteristic peaks of PVA and SA were identified in agreement with previous studies (Zhang J. et al., 2022; Khan et al., 2025). A broad band in the range of 3,690–2,990 cm^-1^ is attributed to O–H stretching vibrations. The bands observed at 2,978–2,870 cm^-1^ correspond to asymmetric and symmetric C–H stretching vibrations. The peaks at 1,328 cm^-1^ and 1,089 cm^-1^ are assigned to C–O and C–OH stretching vibrations, respectively. The band at 1,508–1,381 cm^-1^ is associated with C=O stretching, while the bands at 1,336–1,293 cm^-1^, 1,073 cm^-1^, 955–897 cm^-1^, and 852–835 cm^-1^ are attributed to C–H bending, C–O–C stretching, C–H rocking, and C–C stretching vibrations, respectively, confirming the presence of both PVA and SA in the films.

The incorporation of essential oil micelles into PVA–SA bilayer films leads to an increase in the intensity of the O–H stretching band, indicating a modification of crystallinity due to strong hydrogen bonding interactions between the PVA–SA polymer matrix and the phenolic compounds of the essential oil (Bouftou et al., 2025; 2026a). On the other hand, in the presence of bentonite, a decrease in the intensity of the bands at 3,281 cm^-1^, 1,416 cm^-1^, 1,085 cm^-1^, and 839 cm^-1^ is observed compared to the film without bentonite. This behavior can be attributed to a reduction in hydrogen bonding interactions between the hydroxyl groups of PVA, SA, and the phenolic compounds of the essential oil, as well as weaker interactions with the silicate structure of bentonite (Chen et al., 2017). An opposite behavior has been reported in other studies in the presence of bentonite without essential oil, highlighting the effect of essential oil incorporation (Karimi and Wan Daud, 2016; Chen et al., 2017). In contrast, for PVA–SA–EO films incorporating LDH, the intensity of the bands in the range of 3,674–2,992 cm^-1^ (O–H stretching) increases, along with that of the band around 1,600 cm^-1^. These changes indicate a modification in the interactions between the polymer chains (PVA and SA) and the essential oil in the presence of LDH, suggesting that hydrogen bonding interactions are strengthened in the presence of LDH (Kim et al., 2022).

#### 3.5.4 Scanning electron microscopy (SEM)

The surface morphologies of the developed films are presented in Figure 7. The PVA, SA, and PVA–SA bilayer films exhibit smooth and homogeneous surfaces, with some uniformly distributed accumulations attributed to sodium alginate molecules. A similar behavior has been reported in studies using the same polymer combination, confirming the effectiveness of the preparation method in producing bilayer films with improved surface morphology (Madihalli et al., 2025). In the presence of *Nigella sativa* essential oil micelles, the PVA–SA–EO films show a continuous, relatively uniform, and compact surface, with the presence of some aggregates. This indicates a good dispersion of the essential oil within the polymer matrix (Kumar et al., 2021). In contrast, the PVA–SA–EO–BNT films exhibit an irregular surface, characterized by significant aggregation and the appearance of large pores. This behavior is attributed to less homogeneous dispersion and aggregation phenomena in the presence of the essential oil (Chen et al., 2017). On the other hand, films containing LDH display a similar trend but with smaller pore sizes. This can be explained by a more homogeneous dispersion of essential oil micelles and improved interactions with the PVA–SA matrix, promoted by the presence of LDH.

**FIGURE 7:**
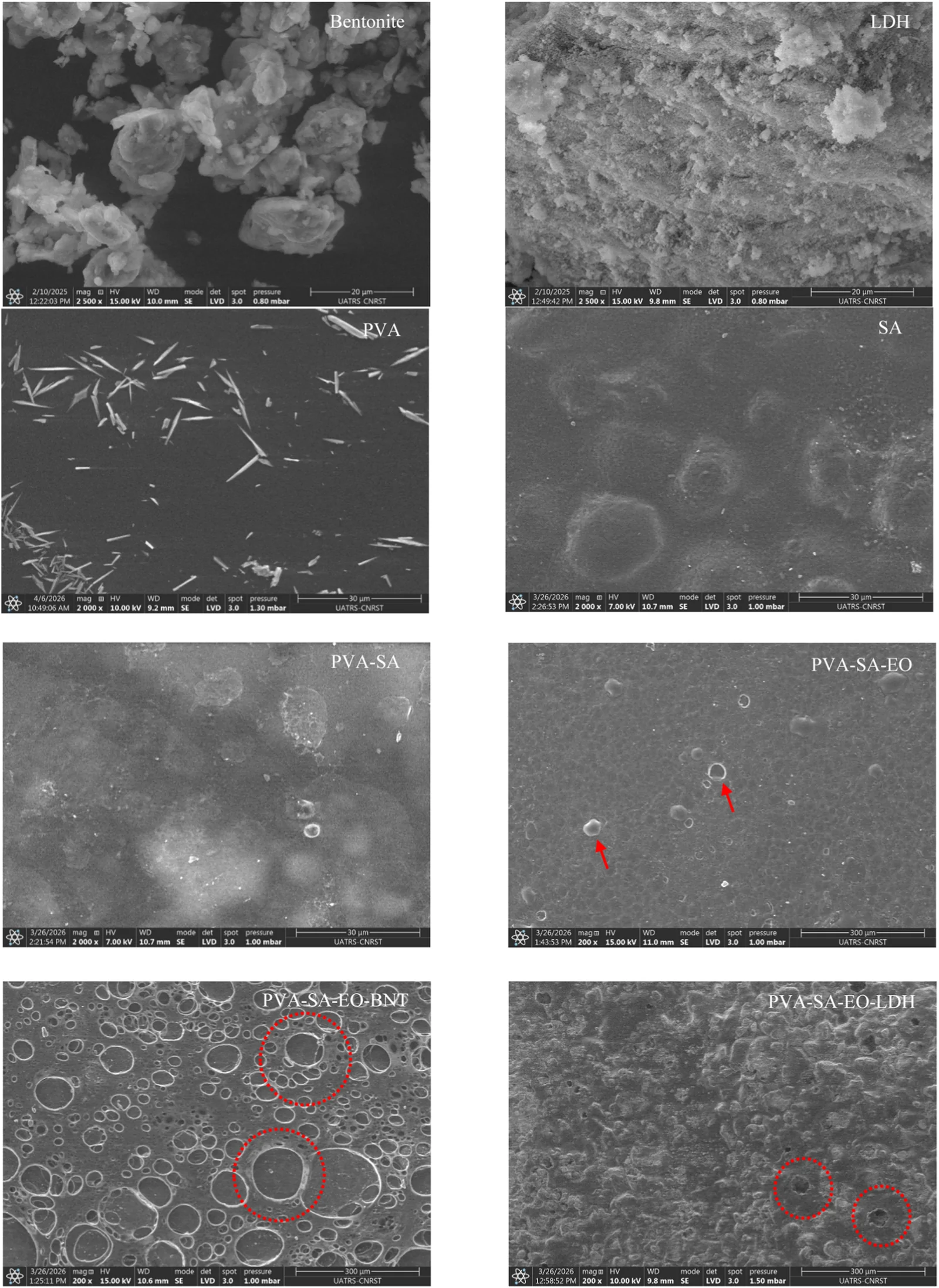
SEM micrographs of Bentonite, LDH, PVA, SA, PVA-SA, PVA-SA-EO, PVA-SA-EO-BNT and PVA-SA-EO-LDH.

#### 3.5.5 X-ray diffraction (XRD)

To investigate the effect of different additives on the crystalline structure of the prepared films, X-ray diffraction (XRD) analysis was performed. The X-ray diffraction patterns of bentonite clay and LDH, presented in Figure 8a, showed that for bentonite clay, the characteristic peaks observed at diffraction angles of 2θ ≈ 19.8°, 26.9°, 35.0°, and 54.7° can be respectively attributed to the crystallographic planes (110), (210) (124), and (144), according to Miller indices (Hebbar et al., 2018). These reflections are in good agreement with the standard JCPDS data (card no. 01–088–0,891), confirming the typical crystalline structure of bentonite. Furthermore, the diffractogram of the Mg-Al LDH matrix highlights the characteristic reflections of layered double hydroxides, particularly the peaks (003), (006) (102), (105) (108), (110), and (113). The basal reflections (003) and (006) are associated with the periodic stacking of the lamellar layers and reflect the interlayer spacing of the material, while the (110) reflection is related to the cationic arrangement within the brucite-like layers. Altogether, these reflections confirm the formation of a Mg–Al LDH phase, characteristic of hydrotalcite-like layered structures (El Meskine et al., 2025).

**FIGURE 8:**
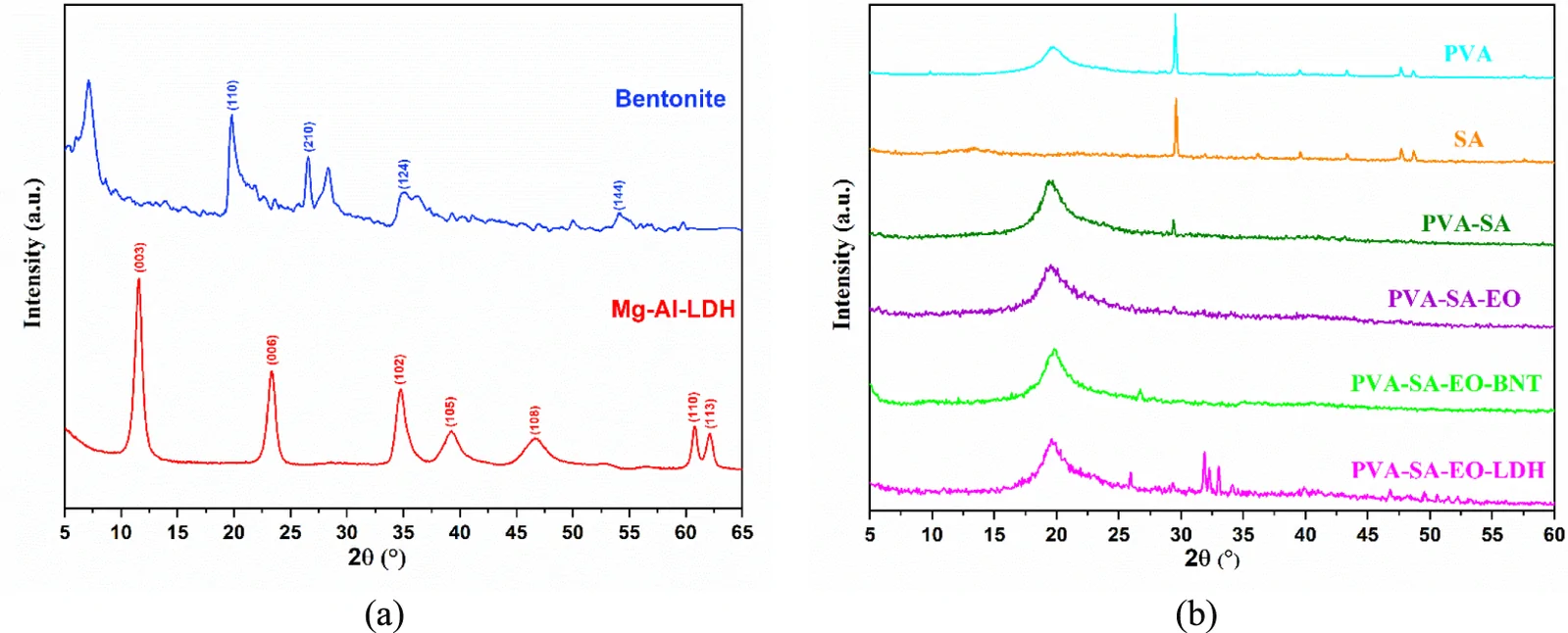
XRD patterns of the clays **(a)** and PVA-SA bi-layer films **(b)**.

The X-ray diffraction (XRD) patterns of the prepared PVA–SA bilayer films are presented in Figure 8b in order to evaluate the structural modifications undergone by these films. The diffraction patterns of all PVA-based films exhibit a characteristic peak around 2θ = 19.8° and another peak around 2θ = 28.5°, which are associated with the semi-crystalline nature of PVA (Voronova et al., 2015; Chen et al., 2017). These results indicate that PVA-based films retain their crystalline structure even in the presence of *Nigella sativa* essential oil and clays. The SA film shows a single diffraction peak at 2θ = 28.5°, characteristic of the amorphous structure of sodium alginate (Miao et al., 2025). Furthermore, the PVA–SA bilayer films display a broadening of the characteristic diffraction band of PVA along with the disappearance of the peak at 2θ = 28.5°, indicating a reduction in crystallinity. This behavior is attributed to strong intermolecular hydrogen bonding interactions between PVA and SA, leading to the formation of a more amorphous complex. These findings are consistent with the results obtained from other analyses (FTIR and DSC) as well as with several reports in the literature (Dinari and Nabiyan, 2017; Kim et al., 2022). With the incorporation of essential oil micelles, either free or in the presence of bentonite or LDH, the diffraction band at 2θ = 19.8° becomes further broadened, suggesting an increase in intermolecular interactions within the matrix. In the presence of bentonite, the diffraction patterns show the absence of the characteristic peaks of this clay in the corresponding films. This behavior can be explained by the exfoliation of bentonite silicate layers within the polymer matrix, resulting in an increased interlayer spacing and loss of parallel stacking (Chen et al., 2017). Similar behavior has been reported in other studies involving bentonite-based films (Breen et al., 2012). In contrast, in the presence of LDH, the diffraction pattern shows the disappearance of the characteristic peaks around 2θ = 11° and 2θ = 23°, corresponding to the (003) and (006) reflection planes, respectively, along with the appearance of the (102) reflection peak. This indicates a good dispersion of Mg–Al LDH within the PVA–SA matrix, in agreement with the literature (Kim et al., 2022).

#### 3.5.6 Thermal properties

To investigate the thermal properties of the developed films, DSC and TGA analyses were carried out. Figure 9 presents the DSC and TGA curves of the prepared films. Figure 9a shows the DSC thermograms along with the glass transition temperature (Tg) values of each film. Overall, all films exhibit similar thermal behavior, with slight variations. The DSC curves display two endothermic events: the first, observed between 40 °C and 75 °C, is attributed to the glass transition (Tg), while the second, located between 180 °C and 225 °C, is likely associated with the evaporation of residual water trapped within the polymer chains. Figure 9a indicates that the PVA–SA bilayer film exhibits a higher Tg (52.93 °C) compared to pure PVA (50.93 °C) and SA (49.93 °C) films. This increase can be attributed to strong intermolecular interactions, particularly hydrogen bonding between the two polymers, as previously confirmed by rheological, XRD, and FTIR analyses. In contrast, the incorporation of Nigella sativa essential oil leads to a decrease in Tg to 49.93 °C. This reduction is attributed to the plasticizing effect of the essential oil, which intercalates between polymer chains, thereby weakening intermolecular interactions and increasing chain mobility. Similar behavior has been widely reported in the literature (Marano et al., 2022). Furthermore, the PVA–SA–EO–BNT and PVA–SA–EO–LDH films show a further decrease in Tg to 48.93 °C. This result suggests that, in this system, the clay fillers promote a better dispersion of the essential oil within the polymer matrix, thereby enhancing the plasticizing effect. This behavior contrasts with other studies reported in the literature, where the incorporation of LDH into polymer matrices, such as PVA and cellulose acetate films, led to an increase in Tg due to strong polymer–filler interactions and restricted chain mobility (Kim et al., 2022; Oliviero et al., 2023).

**FIGURE 9:**
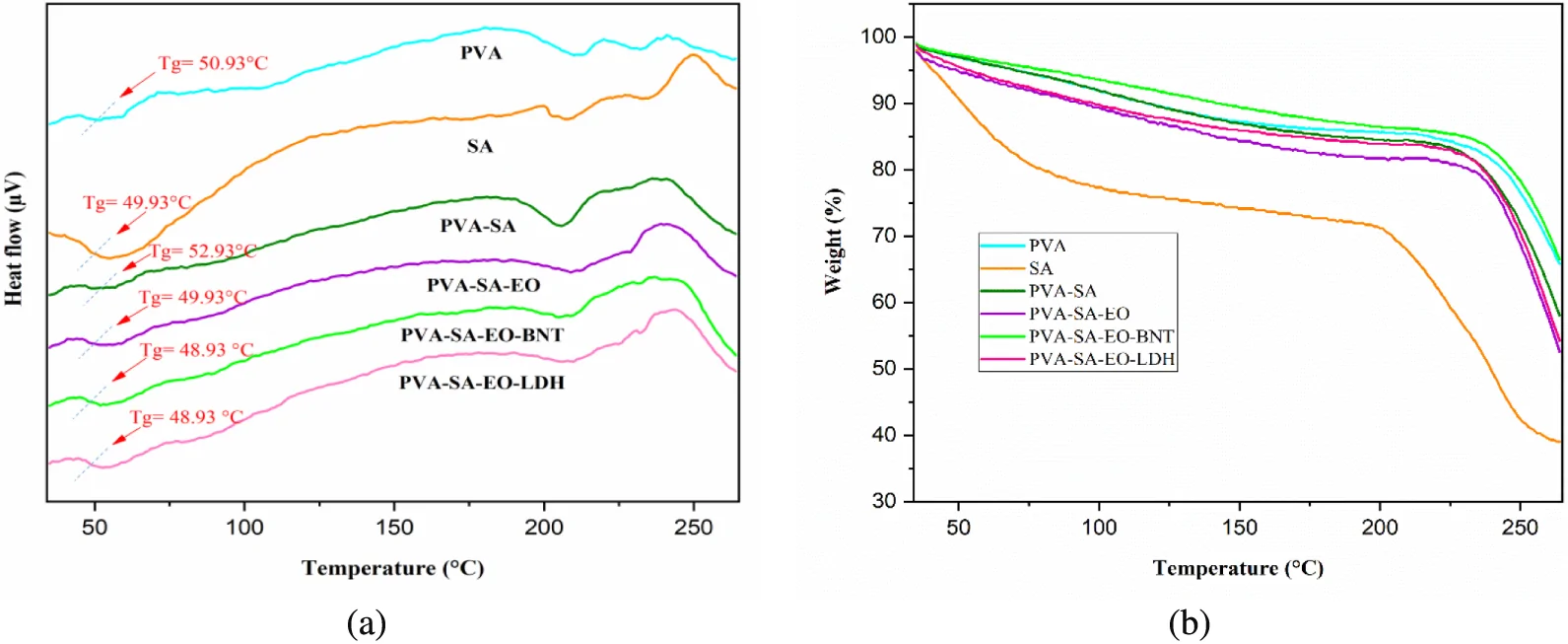
DSC **(a)** and TGA **(b)** thermograms of PVA–SA bilayer films.

The thermogravimetric analysis (TGA) results of the films, presented in Figure 9b, show that the thermal degradation of the PVA-SA film occurs in three main stages. The first stage, observed between 25 °C and 150 °C, is attributed to the evaporation of moisture adsorbed or trapped within the matrix. The second stage, occurring between 200 °C and 300 °C, corresponds to the degradation of the side chains of the polymers. The third stage (≥300 °C) is associated with the main degradation of the PVA and SA polymer backbones (Chen et al., 2017). The TGA results of the PVA-SA-EO films reveal a shift in degradation temperatures toward lower values compared to the PVA–SA film. This behavior can be explained by the low thermal stability of the non-encapsulated essential oil (Bouftou et al., 2024b; 2024c). In contrast, the thermal properties of the PVA-SA-EO-BNT and PVA-SA-EO-LDH films are improved compared to those containing free essential oil. This improvement can be attributed to the enhanced thermal stability of essential oil micelles intercalated within the clay layers, as well as to the formation of aggregates in the presence of clay fillers. These findings are consistent with the results obtained from DSC analysis.

#### 3.5.7 Mechanical properties

The stress–strain curves of the prepared PVA–SA bilayer films are presented in Figure 10a. The obtained results show that the different additives have distinct effects on the mechanical properties of the films. In order to evaluate the combined effect of PVA and SA, as well as the influence of *Nigella sativa* essential oil and the two clays (bentonite and LDH), tensile strength (TS) and elongation at break (EAB) were measured and are presented in Figure 10b. The PVA film exhibits a TS and EAB of 22.808 MPa and 343.177%, respectively, in agreement with the literature, confirming the flexible and semi-crystalline nature of PVA (Thammasang et al., 2026). In contrast, the SA film shows a TS of 45.983 MPa and an EAB of 4.926%, indicating a rigid and brittle material (Ghayour and Ehtesabi, 2026). The PVA–SA bilayer combination leads to an increase in TS to 28.812 MPa and a significant decrease in EAB to 37.095%. This behavior is attributed to strong hydrogen bonding interactions between the two polymers, which restrict chain mobility, in agreement with the FTIR and XRD results (Ghayour and Ehtesabi, 2026). In the presence of *Nigella sativa* essential oil, a significant decrease in TS to 17.780 MPa and an increase in EAB to 161.676% are observed. This behavior is attributed to the plasticizing effect of the essential oil, which enhances polymer chain mobility (Bouftou et al., 2026b; Ghayour and Ehtesabi, 2026). In contrast, films containing bentonite show a marked decrease in both TS and EAB, reaching 10.995 MPa and 7.427%, respectively. This deterioration is attributed to the weakening of intermolecular interactions, as confirmed by FTIR and XRD analyses, as well as to the formation of oil–bentonite aggregates within the matrix, as observed in SEM images. These aggregates act as stress concentration points during stretching, thereby weakening the structural integrity of the films (Tee et al., 2013). Similar behavior has been reported in other studies at high bentonite loadings (Chen et al., 2017). On the other hand, films containing LDH show an increase in TS and EAB to 13.274 MPa and 16.678%, respectively, compared to bentonite-containing films. This relative improvement is attributed to better dispersion of LDH and reduced aggregation in the presence of essential oil. However, the lower TS compared to the PVA-SA-essential oil film remains associated with the formation of microvoids and interfacial debonding, as confirmed by SEM observations (Huang et al., 2011; Kim et al., 2022).

**FIGURE 10:**
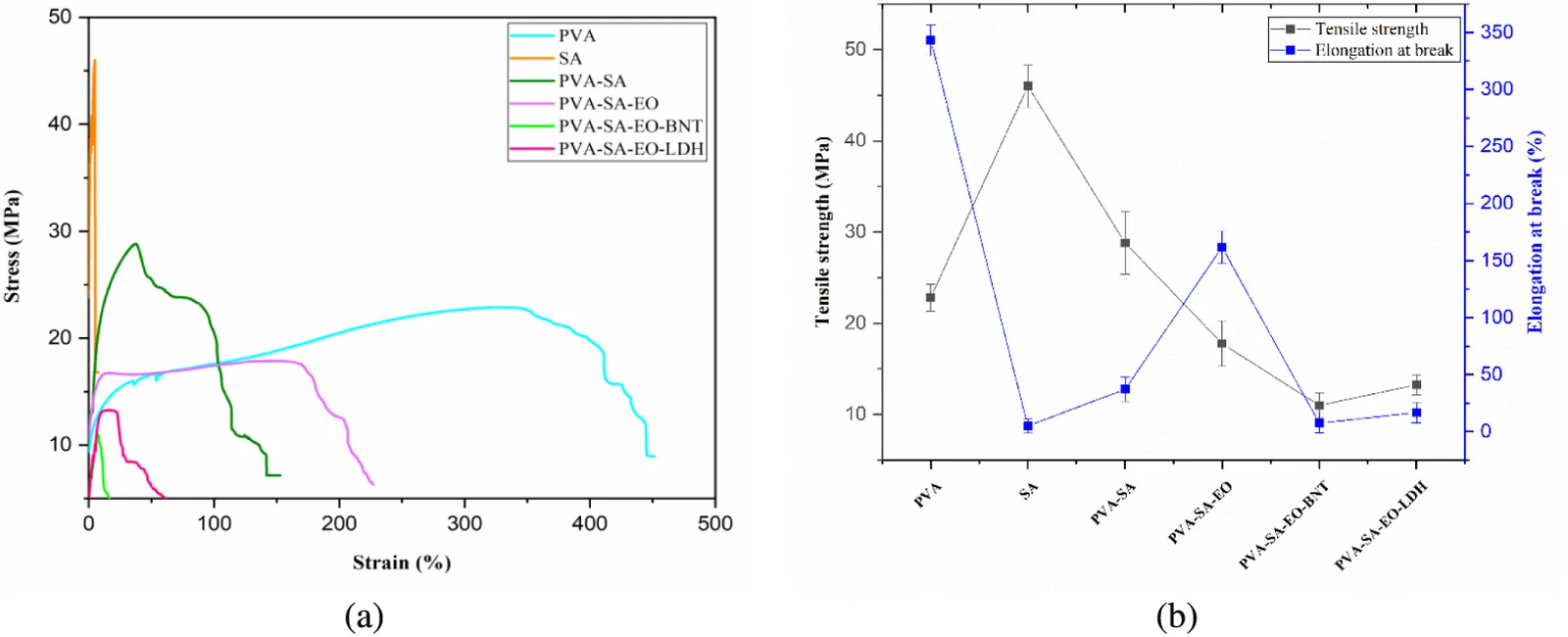
Mechanical properties of the PVA–SA bilayer films. **(a)** Stress–strain curve, **(b)** TS and EAB of the nanocomposite films.

### 3.6 Functional properties of the film

#### 3.6.1 Ultraviolet and visible light transmission

Food packaging films must possess the ability to protect food against oxidative degradation and quality deterioration induced by UV radiation. This property is essential for evaluating the effectiveness of the developed films (Qin et al., 2015). Figure 11 presents the transmittance spectra of the different prepared films in the wavelength range of 200–800 nm. Pure PVA films exhibited the highest light transmittance with almost no absorption, indicating their high transparency. However, this behavior represents a limitation of PVA-based films, as they remain relatively inert toward UV radiation and provide poor UV-barrier properties (Madihalli et al., 2025). In contrast, sodium alginate (SA) films showed lower transmittance in both the visible and UV regions, even when used alone, demonstrating their intrinsic ability to partially block UV radiation. On the other hand, the bilayer PVA-SA films exhibited an intermediate behavior in terms of UV-blocking capacity, with a significantly improved barrier effect compared to pure PVA films, but still lower than that of SA films. The incorporation of Nigella sativa essential oil micelles into the films resulted in a significant decrease in transmittance, which became lower than that of both PVA-SA bilayer and SA films. This notable enhancement in UV-blocking performance can be attributed to the presence of bioactive compounds in Nigella sativa essential oil, as identified by GC-MS analysis (Table 2) (Adilah et al., 2018). These compounds contain electron-rich functional groups capable of interacting with incident light, thereby reducing light transmission through absorption and scattering mechanisms (Amrani et al., 2019).

**FIGURE 11:**
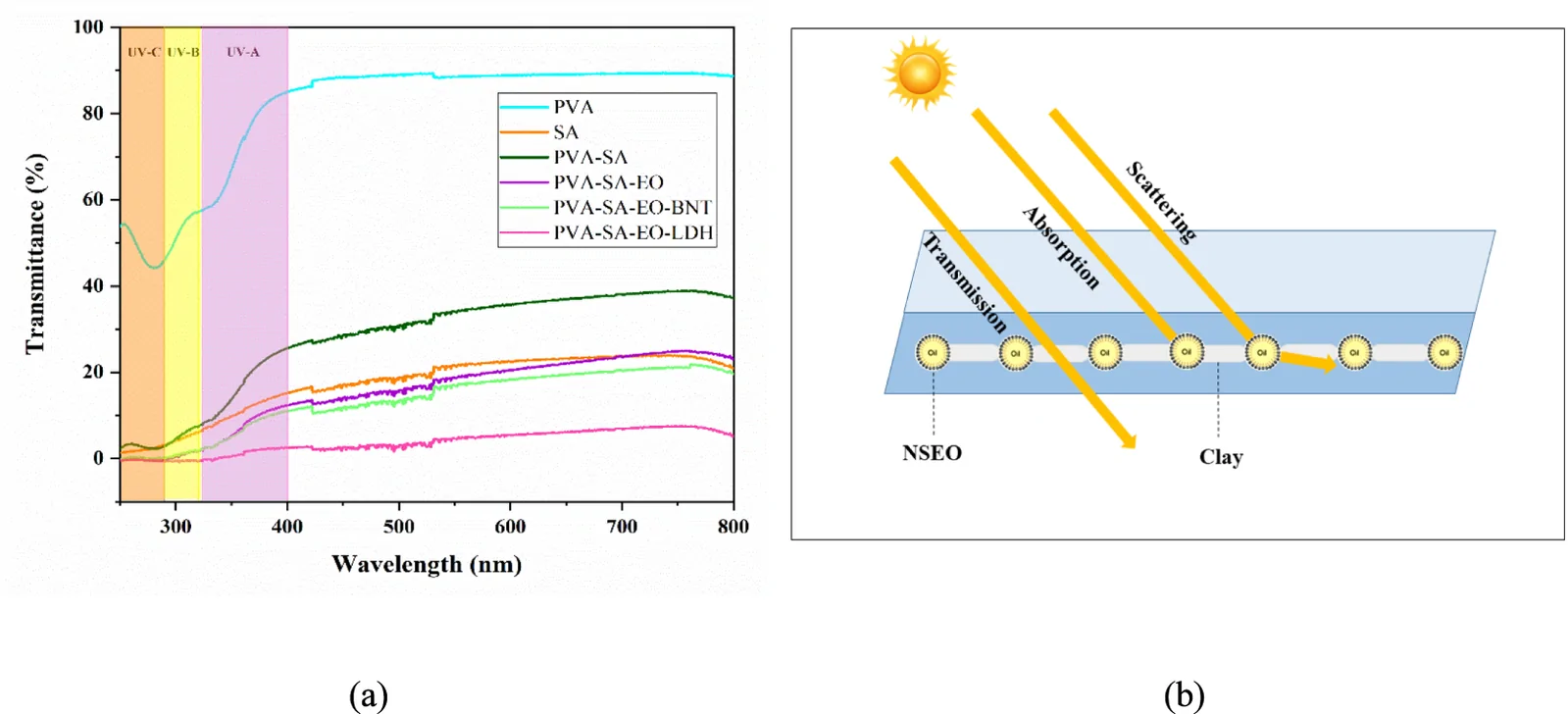
UV-Vis light transmission of PVA-SA bi-layer films **(a)** and schematic diagram of light-blocking principle **(b)**.

Furthermore, the incorporation of clay minerals such as bentonite or Mg-Al LDH in combination with essential oil micelles led to an even more pronounced decrease in transmittance. This behavior can be attributed to the combined effects of light absorption by essential oil components and enhanced light scattering induced by micelles and inorganic fillers. The presence of these particles increases structural heterogeneity and creates a more complex optical pathway, thereby improving the UV-barrier properties of the films (Figure 11b). Notably, Mg-Al LDH exhibited a stronger UV-blocking effect compared to bentonite, which can be explained by its better dispersion within the polymer matrix, in agreement with the SEM images (Figure 7), as well as its well-ordered lamellar structure and stronger interactions with the encapsulated compounds.

#### 3.6.2 Water vapor transmission rate (WVTR)

The water vapor barrier properties of the developed films were evaluated by measuring the water vapor transmission rate (WVTR), and the results are presented in Figure 12a. In general, previous studies have shown that the WVTR of polymeric films decreases in the presence of clay fillers (Guo et al., 2009; Cui et al., 2015). The WVTR values of PVA, SA, and PVA–SA films were 50, 100, and 32 g m^-2^·h^-1^, respectively. These results are consistent with the intrinsic properties of the polymers: PVA is a hydrophilic polymer with moderate permeability, whereas SA exhibits a more open structure, leading to higher permeability. The combination of PVA and SA significantly reduces the WVTR due to the formation of hydrogen bonds between the two polymers, resulting in a more compact and less permeable structure (Madihalli et al., 2025). The incorporation of *Nigella sativa* essential oil led to a significant increase in WVTR, reaching 48 g m^-2^·h^-1^. This increase can be explained by the formation of microcavities within the polymer matrix, caused by the disruption of interactions between PVA and SA (Bouftou et al., 2024a). Furthermore, the addition of bentonite and LDH slightly reduced the WVTR to 46 and 45 g m^-2^·h^-1^, respectively. These results suggest a relatively good dispersion of the lamellar nanoparticles within the PVA–SA–EO films, leading to the formation of tortuous pathways that hinder the diffusion of water molecules (Figure 12b) (Yu et al., 2020; Kim et al., 2022). However, the barrier properties remain lower than those of the PVA–SA film. This decrease may be attributed to the weakening of intermolecular interactions (particularly hydrogen bonding and possibly ester linkages) between the polymer matrices, as well as to potential particle aggregation and morphological heterogeneity. These observations are supported by the SEM images.

**FIGURE 12:**
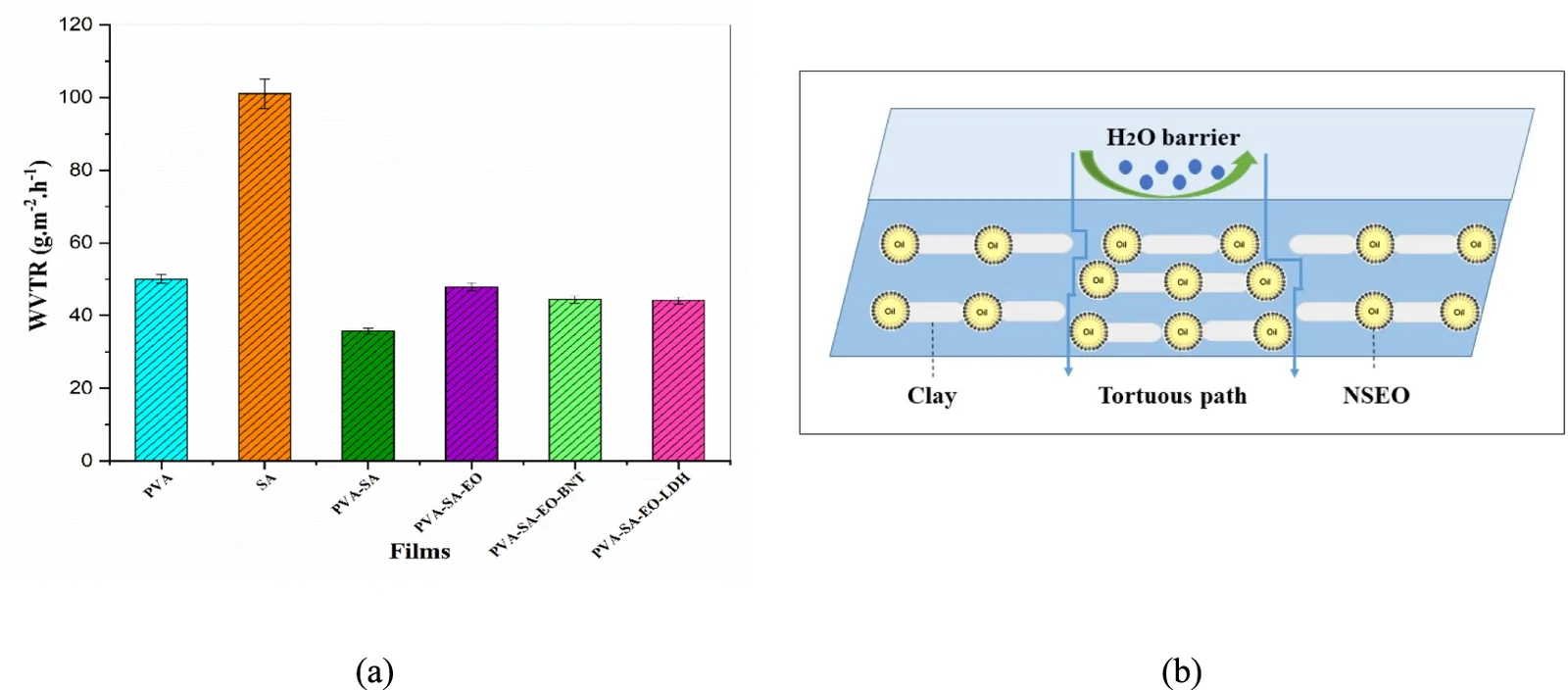
Water vapor transmission rate (WVTR) of PVA-SA bi-layer films **(a)** and schematic representation of the water vapor barrier mechanism in the PVA-SA-EO-clay films **(b)**.

#### 3.6.3 Antioxydant activity

The antioxidant activity of the prepared films was evaluated using the DPPH radical scavenging assay, and the results are presented in Figure 13. The control films (PVA, SA, and PVA–SA) exhibited DPPH scavenging activities of 20%, 24.5%, and 28%, respectively, indicating the limited intrinsic antioxidant activity of both polymers (Yu Y. et al., 2026). In contrast, the films incorporating *Nigella sativa* essential oil showed a significant increase in antioxidant activity, reaching 59%. This improvement is mainly attributed to the synergistic effect between the hydroxyl groups of the PVA and SA matrices and those of the phenolic compounds present in the essential oil, which are capable of donating hydrogen atoms to neutralize free radicals (Akhouy et al., 2024; Yu Y. et al., 2026). Furthermore, films reinforced with clay fillers showed a slight decrease in antioxidant activity. This reduction is attributed to the entrapment of bioactive compounds from the essential oil within the clay structures, which reduces their availability. However, despite this decrease, the PVA–SA–EO–BNT and PVA–SA–EO–LDH films still exhibit higher antioxidant activity than the control films.

**FIGURE 13:**
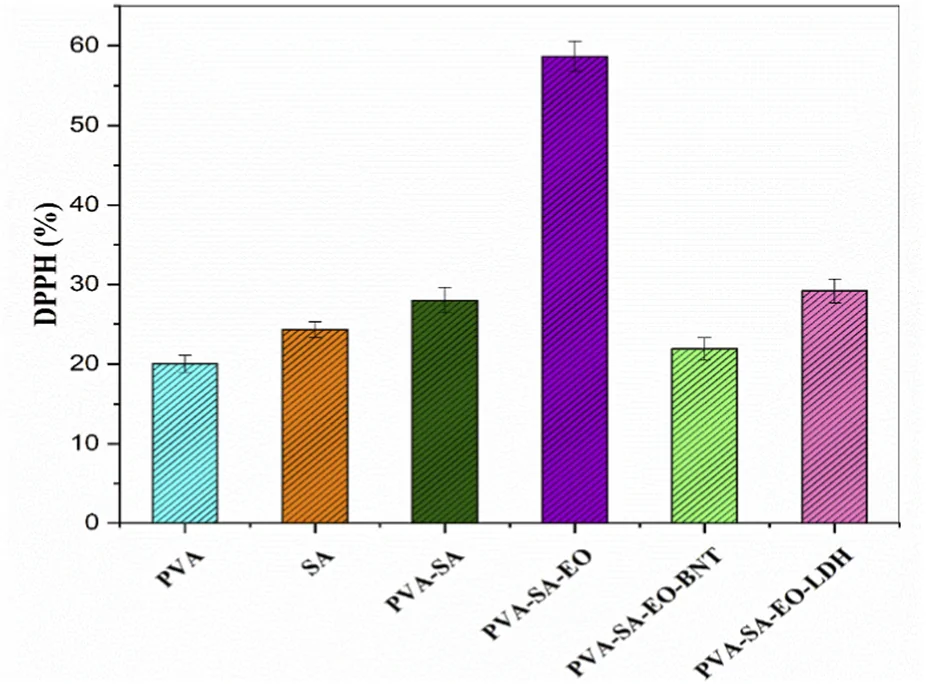
DPPH scavenging activity of PVA-SA bi-layer films.

#### 3.6.4 Antibacterial activity

The antibacterial activities of the prepared films were evaluated using the disk diffusion method against *Escherichia coli* infection (*E. coli*, Gram-negative bacterium) and *Staphylococcus aureus* infection (*S. aureus*, Gram-positive bacterium). These two microorganisms were selected because they are among the major bacteria responsible for food spoilage (Tang et al., 2019). The inhibition zone diameters are presented in Figure 14a. According to the obtained results, the control films (PVA, SA, and PVA–SA) exhibited weak antibacterial activity, with inhibition zone diameters against *S. aureus* of 8 mm, 9 mm, and 9.2 mm, respectively, and 8 mm, 9.2 mm, and 9.4 mm against *E. coli*, respectively. Furthermore, the incorporation of *Nigella sativa* essential oil significantly enhanced the antibacterial activity against both microorganisms (p < 0.05), with inhibition zones of 24 mm against *S. aureus* and 23.5 mm against *E. coli*. This improvement is mainly attributed to the rapid release of the non-entrapped essential oil. Indeed, the hydrophobic bioactive compounds of *Nigella sativa* essential oil, rich in phenolic and aldehyde groups, are able to penetrate bacterial cell membranes, disrupt their integrity, induce leakage of intracellular contents, interfere with essential enzymatic functions, and ultimately lead to bacterial cell death (Zhang J. et al., 2022; Yu Y. et al., 2026) (Figure 14b). In contrast, in the presence of clay fillers, either bentonite or LDH, a reduction in the initial antibacterial activity was observed. The inhibition zones decreased to 21 and 20 mm against *S. aureus* and *E. coli*, respectively, for the PVA-SA-EO-BNT films, and to 20.5 and 21 mm, respectively, for the PVA-SA-EO-LDH films. This lower initial activity may be related to the retention of essential oil bioactive compounds within the clay structures, which can reduce their immediate availability at the bacterial interface. This interpretation is consistent with the lower cumulative release observed for the clay-containing films. However, since antibacterial activity was evaluated only after 24 h, the present results cannot confirm whether the slower release provided sustained antimicrobial activity over longer periods. Further time-dependent antibacterial studies would therefore be required to establish a direct relationship between essential oil release and long-term antimicrobial efficacy.

**FIGURE 14:**
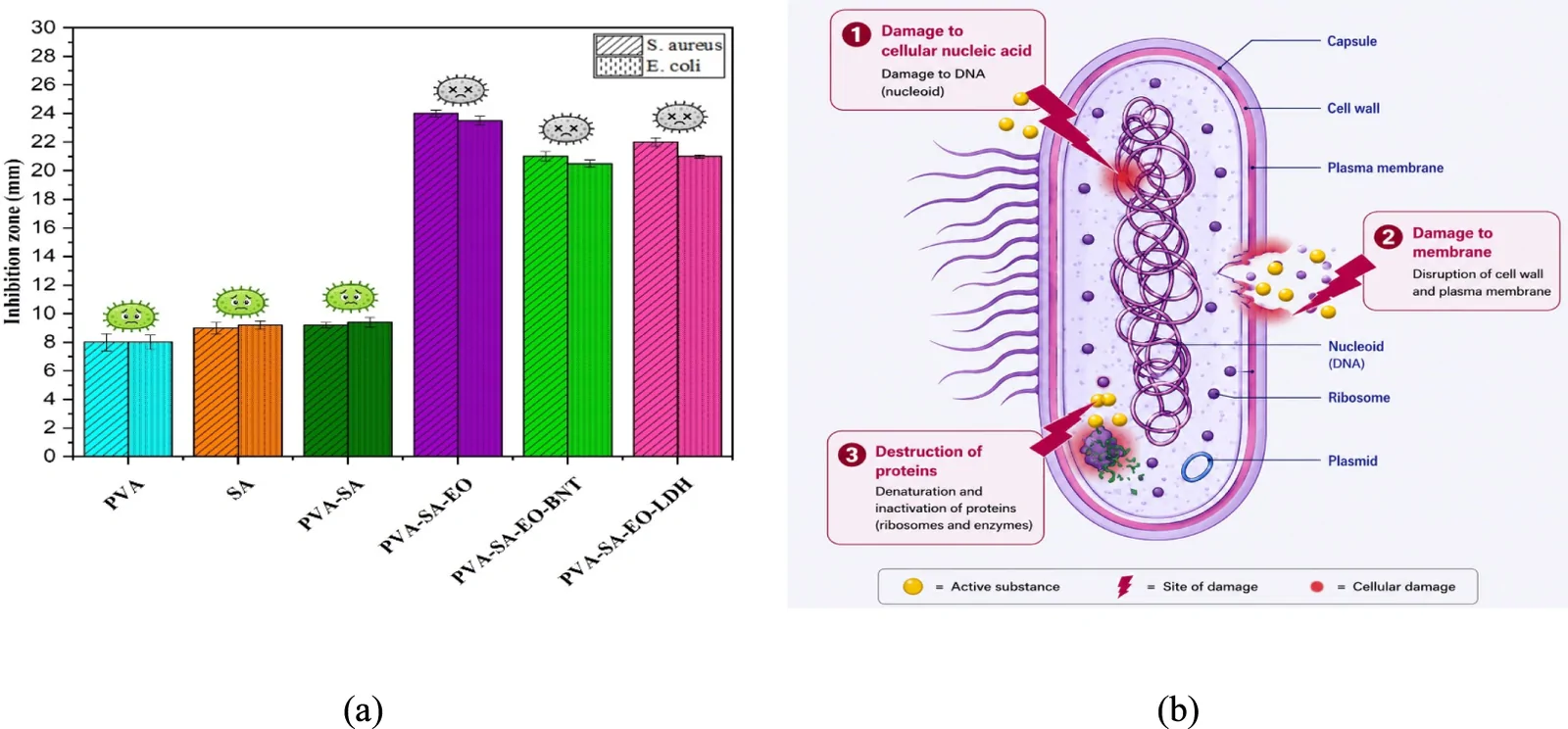
Antibacterial activity of PVA-SA bi-layer films against human pathogenic bacteria **(a)** and antibacterial mechanism diagram of PVA-SA bi-layer coating **(b)**.

#### 3.6.5 Cumulative release behavior in food simulants

The release behavior of *Nigella sativa* essential oil compounds in a DMSO solution used as a food simulant at room temperature was investigated, and the obtained results are presented in Figure 15. The results showed that the PVA–SA–EO films gradually released the essential oil, with a release rate not exceeding 20% during the first 6 days. After the sixth day, the non-encapsulated essential oil exhibited a much faster release profile, reaching 100% after 14 days. These findings indicate that the PVA–SA bilayer films are capable of stabilizing the essential oil only for a limited period of approximately 6 days, after which the oil diffuses completely in an uncontrolled manner, Similar behavior has been widely reported in the literature (Saucedo-Zuñiga et al., 2021; Zhang T. et al., 2022; Bouftou et al., 2024b). In contrast, the films containing essential oil in the presence of the two clay fillers exhibited a similar release behavior, but with an extended stabilization phase lasting up to 8 days. Thereafter, the release rate gradually increased, reaching 28% in the presence of bentonite and 80% in the presence of LDH after 14 days. These results clearly confirm the significant effect of using clay materials, particularly bentonite and LDH, in improving the stabilization of *Nigella sativa* essential oil and controlling its release within the polymer matrix.

**FIGURE 15:**
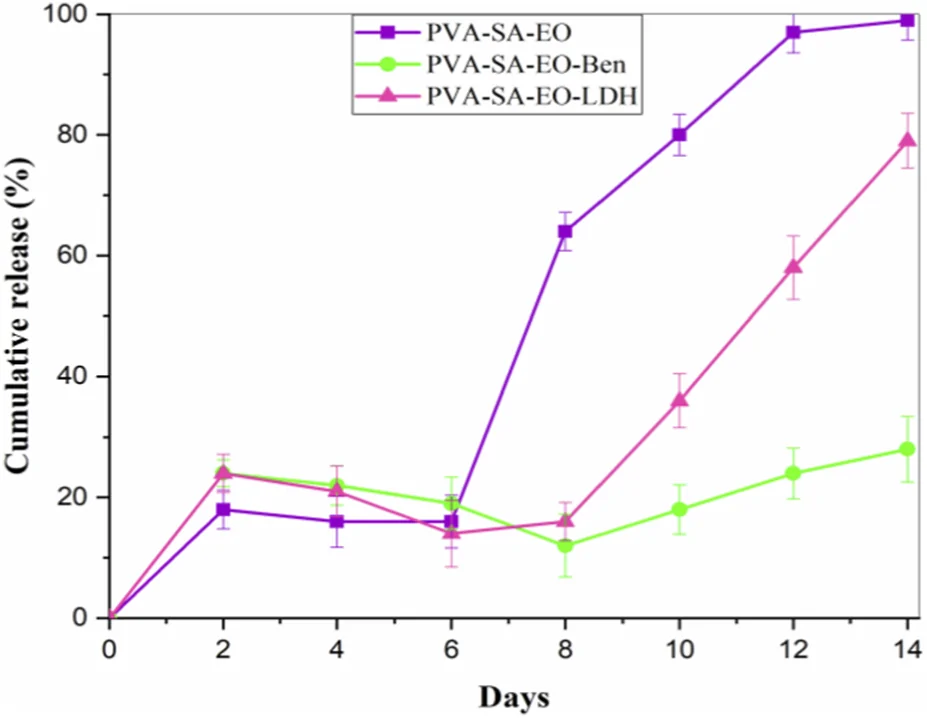
Cumulative release of NSEO from PVA-SA bi-layer films.

### 3.7 Performance of the coatings

#### 3.7.1 Appearance and color parameters

One of the most commonly used parameters by consumers to assess beef freshness is its visual appearance. Among the various quality attributes, color is considered an essential indicator that provides an initial assessment of meat quality and freshness. In general, the characteristic red color of beef mainly depends on myoglobin, the pigment responsible for this coloration, as well as several other factors, including the moisture content at the meat surface, microbial activity, and the redox state of the heme group of this protein (Sánchez et al., 2023; Yan et al., 2024). For this reason, the colorimetric parameters L*, a*, and b* of the different beef samples were measured every 2 days during a 10-day storage period. The ΔE values, calculated from these parameters, are presented in Figure 17a. As illustrated in the figure, the ΔE values significantly increased for all samples throughout storage, indicating that the color of the beef progressively deviated from its initial color observed on the first day (Mao et al., 2023). After 10 days of storage, the ΔE values of the control, PVA-SA-EO-coated, PVA-SA-EO-BNT-coated, PVA-SA-EO-LDH-coated, and PE-packaged samples were 13.09, 5.11, 7.48, 3.26, and 9.90, respectively. These results clearly demonstrate that the samples coated with the PVA-SA-based formulations exhibited significantly lower ΔE values than both the control and the PE-packaged samples, indicating a better preservation of beef color. These results are consistent with the images of the samples taken daily over 10 days (Figure 16). The observed trends can be attributed to the antioxidant, antimicrobial, and barrier properties of the PVA-SA coating films containing *Nigella sativa* essential oil and stabilized with either bentonite or layered double hydroxides (LDH). These properties help limit myoglobin oxidation, microbial proliferation, and the transfer of moisture and oxygen, thereby contributing to the preservation of the visual quality and freshness of beef during storage.

**FIGURE 16:**
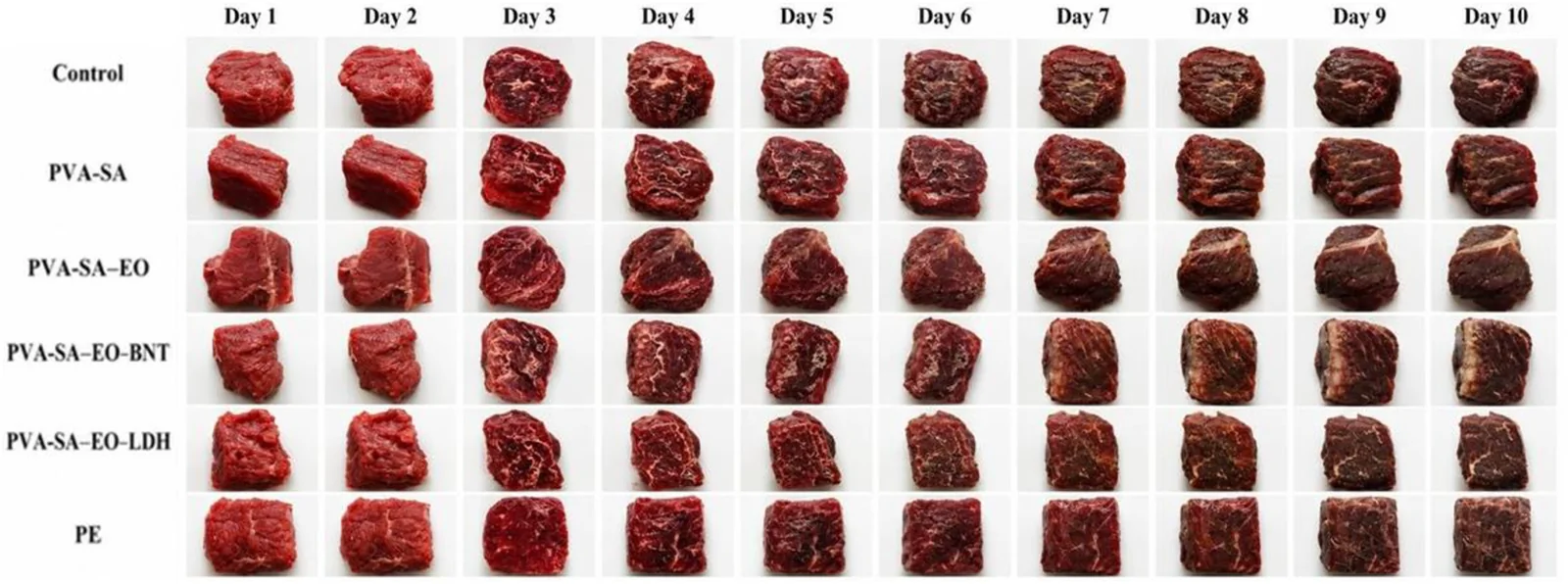
The appearance changes of beef under different treatments at 4 °C for 10 days.

#### 3.7.2 pH

The measurement of pH is considered one of the essential techniques for evaluating beef quality due to its influence on protein stability. The pH values of all tested beef samples are presented in Figure 17b. The results showed that the pH of beef increased to varying degrees during the first 4 days of storage. This increase can be attributed to the partial degradation of proteins into alkaline compounds, particularly ammonia. These alkaline compounds are generally released as a result of biochemical reactions catalyzed by two types of enzymes: endogenous enzymes naturally present in beef and exogenous enzymes produced by microorganisms (Wang et al., 2020). Moreover, a decrease in pH was observed on the sixth day of storage. This behavior can be explained by the dynamic balance between the release of alkaline compounds and the temporary production of acidic metabolites, as well as lipid oxidation phenomena. Subsequently, all samples exhibited another increase in pH, reaching 8.95 for the unpackaged beef and 8.84 for the beef coated with the PVA-SA film after 10 days of storage. In contrast, the other beef samples treated with the different coatings maintained significantly lower pH values compared to the control sample. These results clearly demonstrated that coating beef with our formulations significantly inhibited the increase in pH due to the antimicrobial effect of *Nigella sativa* essential oil stabilized within the coating films in the presence of clays. This antimicrobial activity limited bacterial proliferation in the meat and consequently slowed down the degradation of proteins into alkaline compounds. Similar behavior has also been reported in several studies involving other types of food coatings (Han et al., 2014; Yan et al., 2024).

**FIGURE 17:**
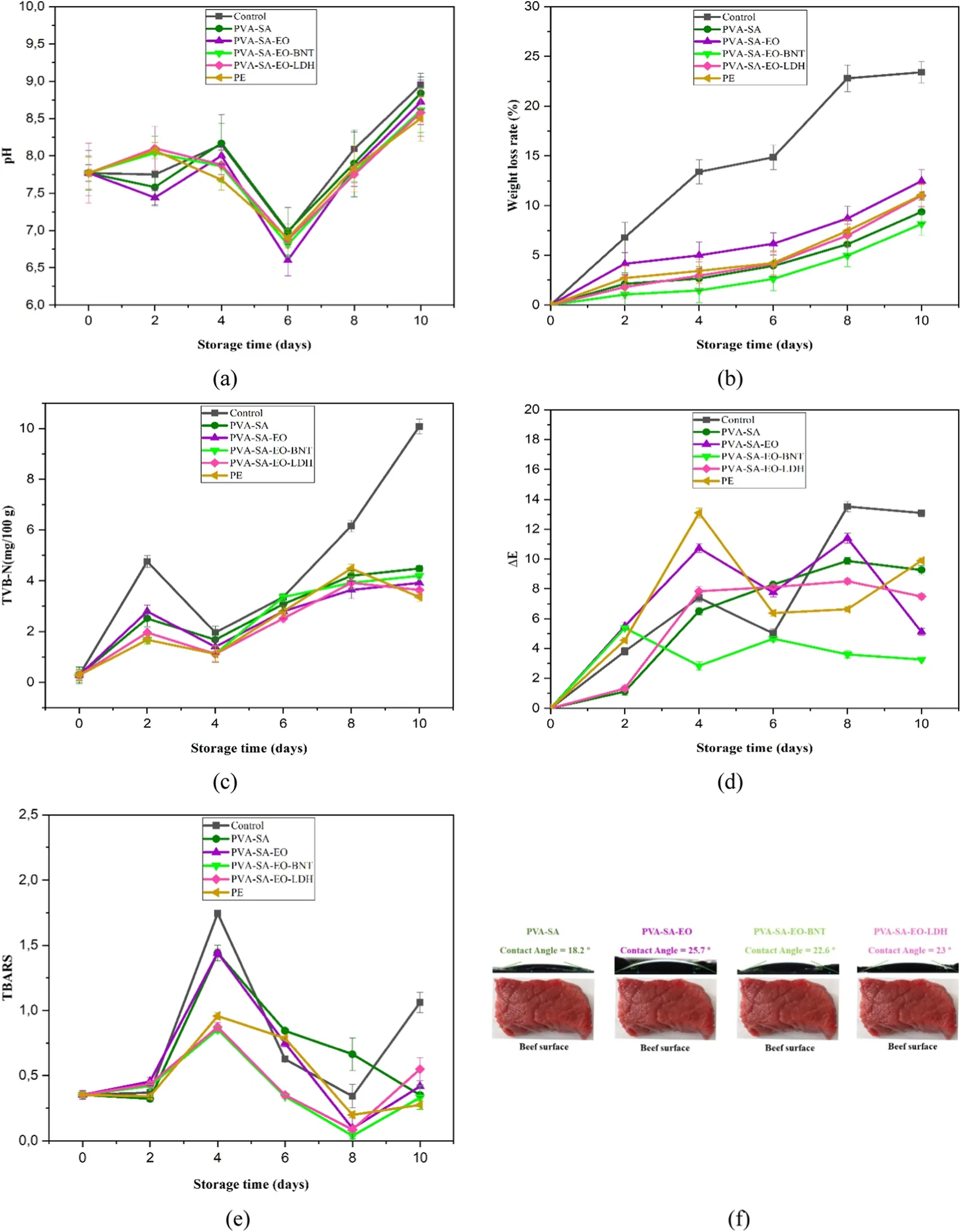
Evolution of beef quality attributes during storage at 4 °C for 10 days: **(a)** color difference (ΔE); **(b)** pH values; **(c)** weight loss rate (%); **(d)** thiobarbituric acid reactive substances (TBARS, mg MDA/kg); **(e)** total volatile basic nitrogen (TVB-N, mg/100 g) and **(f)** Contact angle measurement of coating spreadability on beef surfaces.

#### 3.7.3 Weight loss

Beef stored at 4 °C naturally undergoes weight loss mainly due to water evaporation, which consequently leads to the loss of water-soluble vitamins and sarcoplasmic proteins (Odila Pereira et al., 2018). The results of the weight loss measurements of beef after 10 days of storage at 4 °C are presented in Figure 17c. The unpackaged control samples exhibited the highest weight loss, reaching 23.4%, whereas beef samples packaged either with polyethylene (PE) or coated with our developed formulations showed significantly lower weight loss rates. The lowest value was observed for beef coated with the PVA-SA-EO-LDH film, with a weight loss of 8.17%, while beef samples coated with the PVA-SA-EO-BNT film or packaged with PE exhibited weight losses of approximately 11%. These results clearly demonstrate the effectiveness of the developed coatings, which possess good barrier properties that limit water migration, in agreement with the previously obtained results for water vapor permeability.

#### 3.7.4 TBARS

To evaluate the degree of lipid oxidation in beef, the TBARS index was measured, as this parameter increases with the formation of cytotoxic compounds, particularly malondialdehyde (MDA) (Mahdi et al., 2023). The variations in TBARS values of the control samples, samples coated with our formulations, and samples packaged with polyethylene (PE) are presented in Figure 17d. As shown in the figure, all samples exhibited a significant increase in TBARS values during the first 4 days of storage. The highest TBARS values were observed for the control group, reaching 1.744 mg MDA/kg, compared to the treated samples. This increase can be attributed to the accumulation of degradation products resulting from the decomposition of lipid hydroperoxides and peroxides generated during the oxidation of unsaturated fatty acids (Liu et al., 2026). From the fourth to the eighth day of storage, a decrease in TBARS values was observed. This reduction may be explained by the evaporation of certain volatile substances produced during lipid oxidation, as well as by the interaction of malondialdehyde with proteins and other meat constituents. Subsequently, another increase in TBARS values was observed after the eighth day, indicating a significant resumption of lipid oxidation reactions. On the 10th day of storage, the TBARS values of beef samples coated with PVA-SA-EO-BNT and PVA-SA-EO-LDH films were 0.331 mg MDA/kg and 0.549 mg MDA/kg, respectively, whereas the control sample reached 1.061 mg MDA/kg. These results clearly demonstrate that PVA-SA-based coatings activated with *Nigella sativa* essential oil and stabilized with clays could delay lipid oxidation and consequently improve the shelf life of beef. Similar behavior has been reported in several studies highlighting the effectiveness of essential oils in reducing lipid oxidation (Shin et al., 2017; Mahdi et al., 2023; Yan et al., 2024).

#### 3.7.5 TVB-N

During beef spoilage, alkaline nitrogenous compounds, particularly ammonia and amines, are produced as a result of protein and amino acid degradation. These compounds constitute the main components of total volatile basic nitrogen (TVB-N). Therefore, TVB-N content is widely used as an indicator of meat freshness and the degree of spoilage (Singh et al., 2018). Figure 17e illustrates the effect of the different treatments on the evolution of TVB-N values in beef samples stored at 4 °C. The initial TVB-N value was 0.28 mg/100 g for all samples. The relatively low initial TVB-N values observed in the present study compared with some values reported in the literature may be related to differences in the initial freshness and handling conditions of the beef samples, as well as differences in analytical procedures and the expression of TVB-N results. Therefore, direct comparison among studies should be made with caution (Holman et al., 2021). Nevertheless, the progressive changes observed during storage provide a useful indication of the evolution of beef freshness under the different coating treatments. During storage, TVB-N values increased in all groups, reflecting the progression of spoilage processes. However, this increase was significantly lower in samples coated with our PVA-SA-based formulations and in those packaged with polyethylene (PE) compared to the unpackaged control sample. On the 10th day of storage, the TVB-N values of the control, PVA-SA-EO-coated, PVA-SA-EO-BNT-coated, PVA-SA-EO-LDH-coated, and PE-packaged samples were 10.8, 3.92, 4.20, 3.64, and 3.36 mg/100 g, respectively. These results can be attributed to the effectiveness of the PVA-SA-based coatings activated with *Nigella sativa* essential oil and stabilized with either bentonite (BNT) or layered double hydroxides (LDH). These coatings act as effective barriers against moisture and oxygen while exhibiting antimicrobial activity capable of inhibiting bacterial growth. Consequently, the degradation of proteins and amino acids is slowed down, thereby limiting the formation of volatile nitrogenous compounds responsible for the increase in TVB-N values. These findings are consistent with several previous studies that have demonstrated the effectiveness of natural extracts and essential oils in slowing the accumulation of TVB-N and extending the shelf life of meat products (Cai et al., 2025; Anthomelides et al., 2026; Hashemi et al., 2026).

#### 3.7.6 Contact angle on beef surface

Contact angle is one of the essential parameters used to evaluate coating quality. For this reason, this measurement was performed on beef samples, as shown in Figure 17f The contact angle on the beef surface increased to 25.7° in the presence of *Nigella sativa* essential oil compared to 18.2° for the PVA-SA polymeric solution. This increase is attributed to the hydrophobic nature of the incorporated essential oil. On the other hand, a decrease in the contact angle was observed in the presence of bentonite or LDH, reaching values of 22.6° and 23°, respectively. This indicates that although the essential oil increases surface hydrophobicity, the incorporation of nanoclays slightly reduces this effect due to their hydrophilic nature (EL MESKINE et al., 2026). However, the obtained contact angle values remain within the optimal range for spray-applied coatings, ensuring uniform surface coverage without excessive penetration into the substrate (Bouftou et al., 2026b).

## 4 Conclusion

Active food packaging films based on PVA-SA bilayer structures incorporating *Nigella sativa* essential oil and two types of nanoclays, namely, a natural clay (bentonite, BNT) and a synthetic clay (LDH), were successfully developed. Prior to their incorporation into the films, the chemical composition of the essential oil was characterized by GC-MS analysis, revealing its richness in bioactive phenolic compounds. The nanoclays were characterized by BET analysis, which highlighted significant differences in specific surface area between bentonite and LDH. The essential oil-clay systems dispersed in aqueous media were characterized by optical microscopy, particle size distribution analysis, and zeta potential measurements. The results demonstrated that the presence of the essential oil significantly influenced the interactions between clay particles. Furthermore, molecular docking simulations revealed the existence of intermolecular interactions between the polymer matrices and the major phenolic compounds of the essential oil. These interactions were subsequently confirmed by rheological, FTIR, and XRD analyses. The incorporation of *Nigella sativa* essential oil in the presence of nanoclays imparted high antioxidant and antibacterial activities to the films against both Gram-positive and Gram-negative bacteria. In addition, this incorporation improved the mechanical properties of the films while reducing their water vapor permeability, water solubility, and water absorption capacity. The PVA-SA-EO-clay bilayer films also exhibited enhanced UV-barrier properties and improved surface hydrophobicity. Moreover, they enabled the controlled release of the essential oil bioactive compounds was observed up to 14 days. The functional properties of the developed films were further exploited through their application as spray coatings for the preservation of refrigerated beef stored at 4 °C for 10 days. The coatings demonstrated a strong ability to delay beef deterioration while maintaining its overall quality. In particular, they significantly limited the increases in pH, total volatile basic nitrogen (TVB-N), thiobarbituric acid reactive substances (TBARS), and weight loss compared with untreated control samples. Overall, the obtained results indicate that these active coatings represent a promising and sustainable strategy for preserving the quality of protein-rich foods, extending their shelf life, reducing food waste, and enhancing food safety.

## Data Availability

The original contributions presented in the study are included in the article/supplementary material, further inquiries can be directed to the corresponding authors.
